# Supplementary material for: HIF-1 inactivation empowers HIF-2 to drive hypoxia adaptation in aggressive forms of medulloblastoma
Source: Cell Death Discov. 2024 Jul 24;10:338. doi: 10.1038/s41420-024-02100-5 (PMC11269614; doi:10.1038/s41420-024-02100-5)
Supplement: Supplementary file 1 — Suppl. Figures 1–14 [file 41420_2024_2100_MOESM1_ESM.pdf]

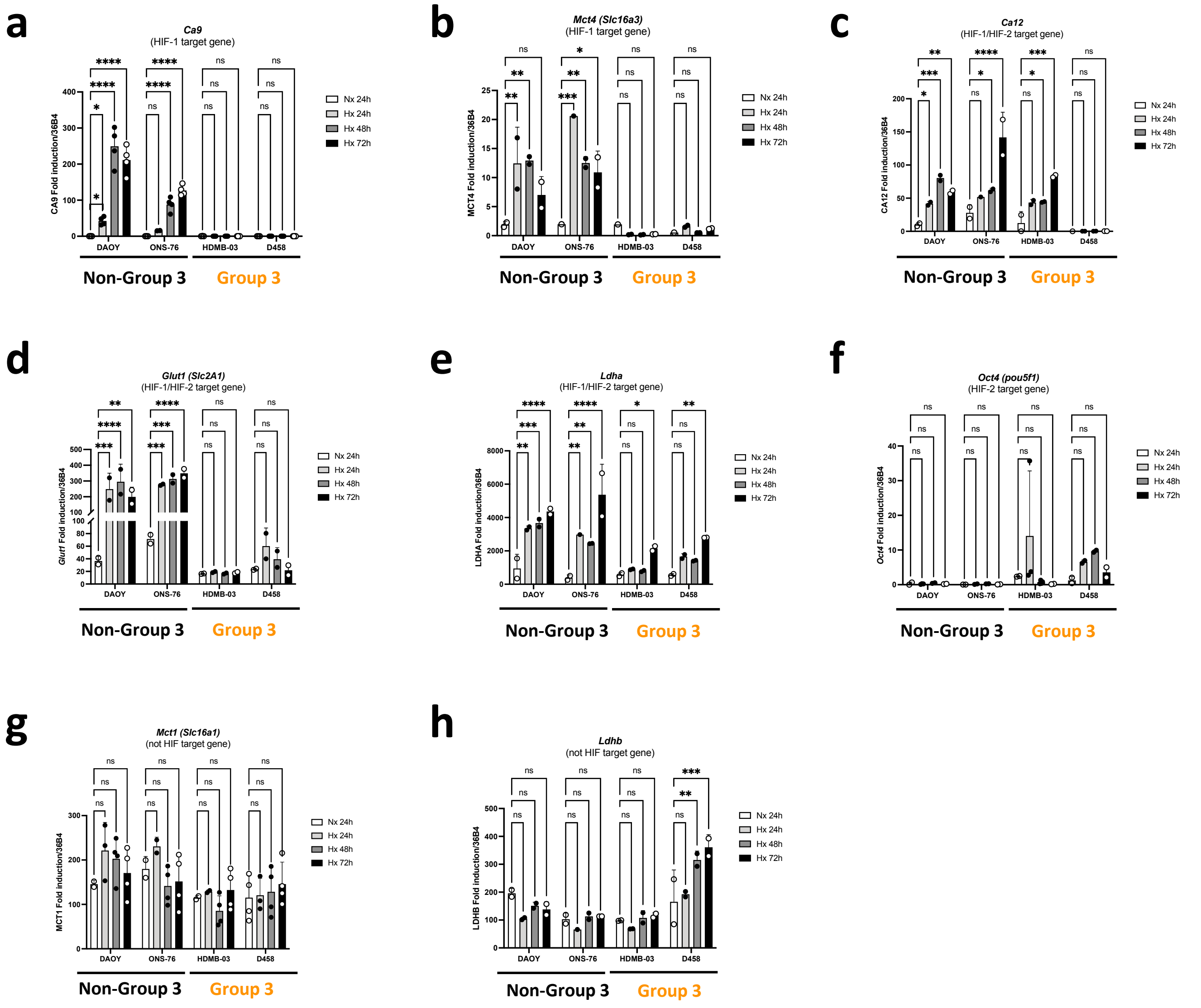

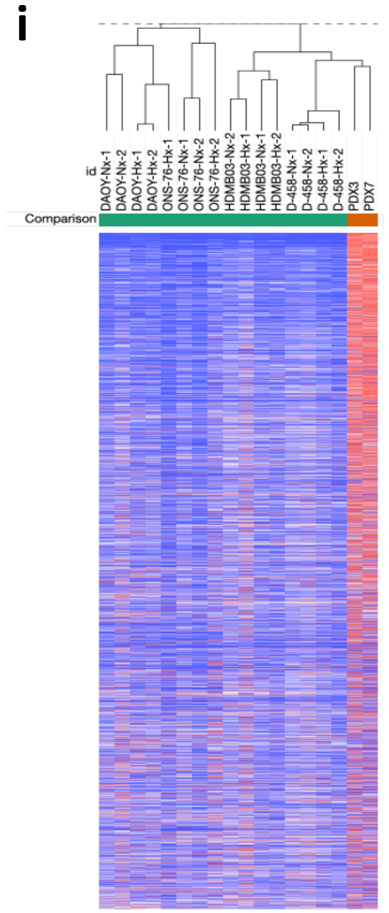

Suppl. FIGURE 1

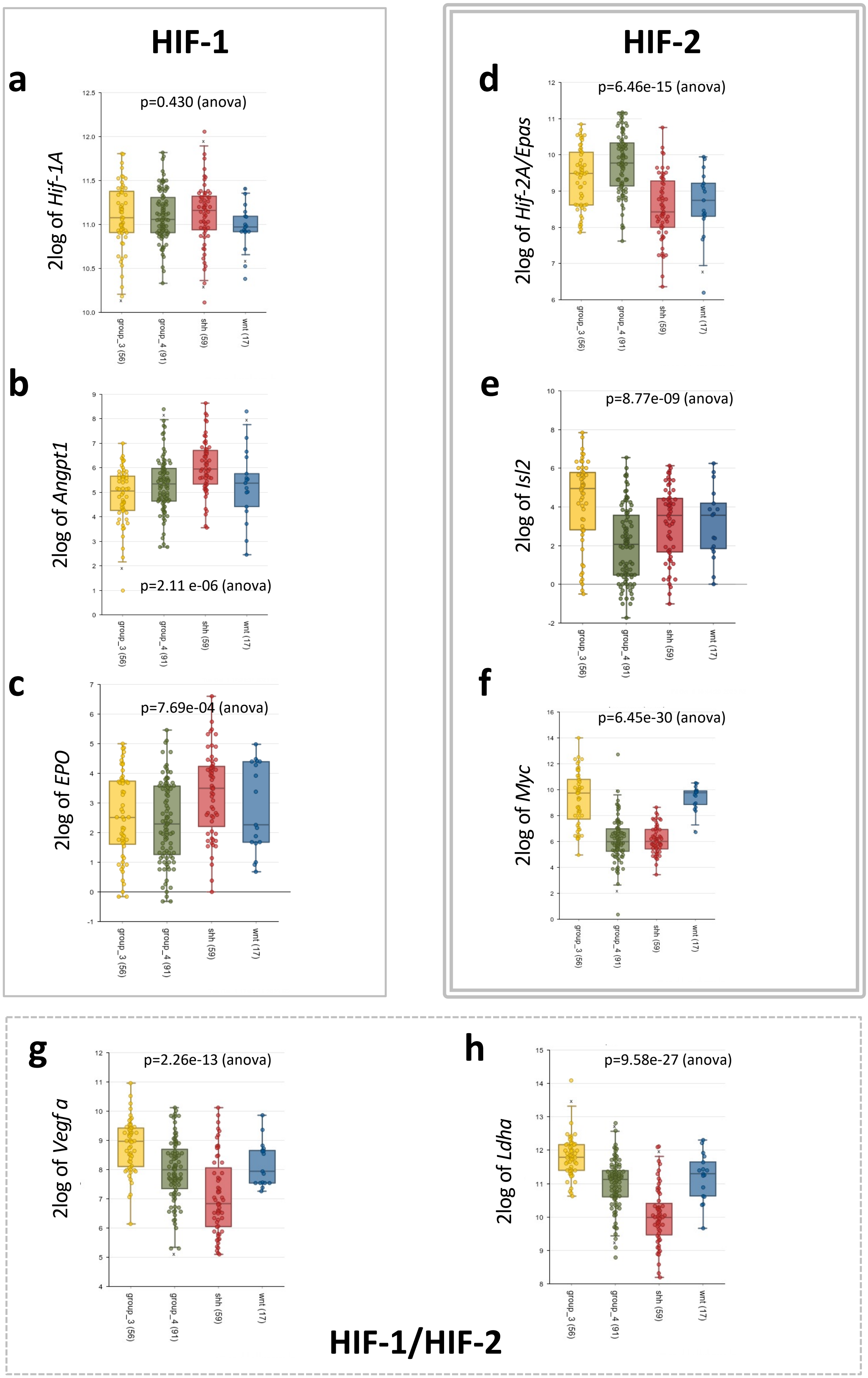

Suppl. FIGURE 2

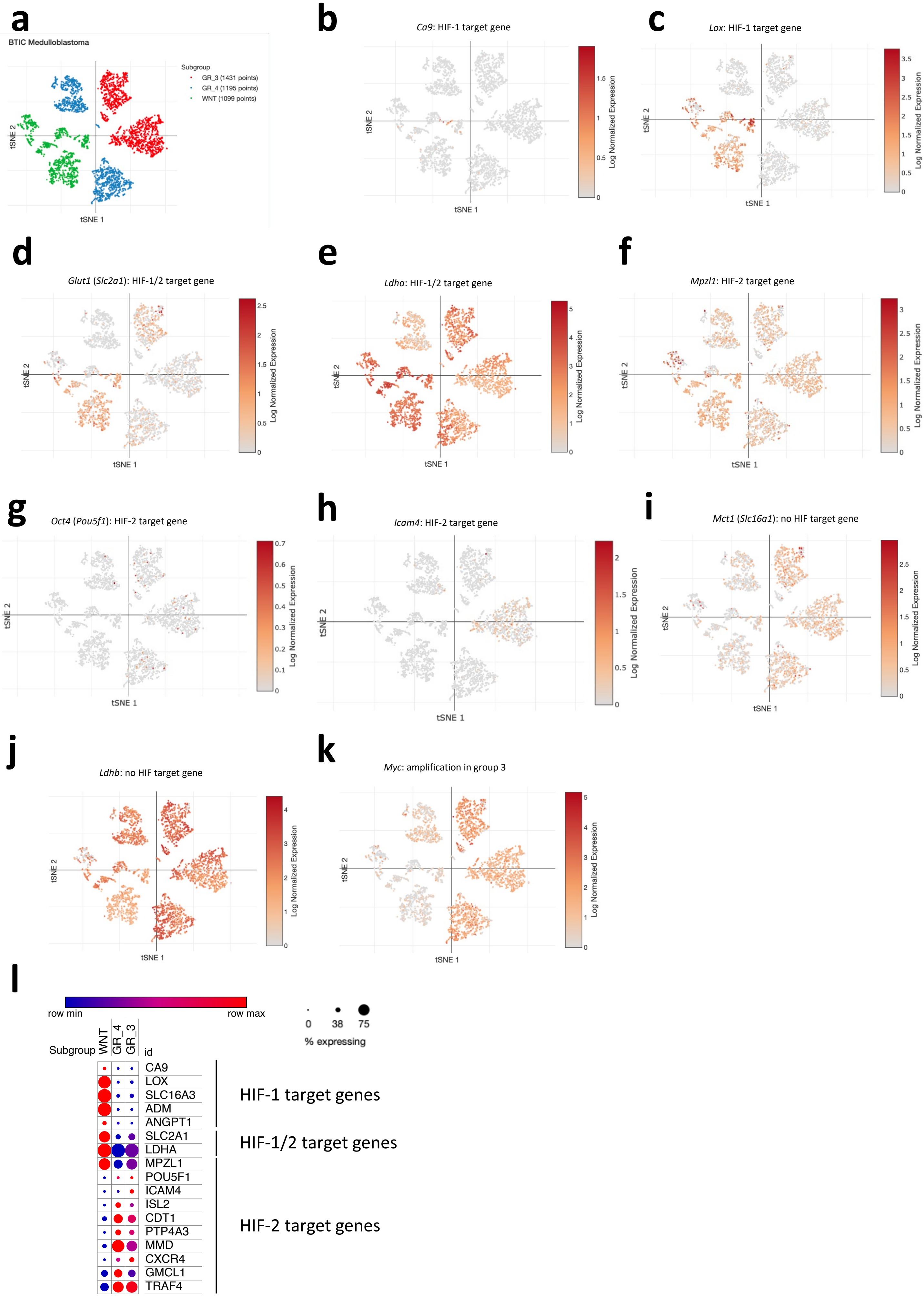

Suppl. FIGURE 3

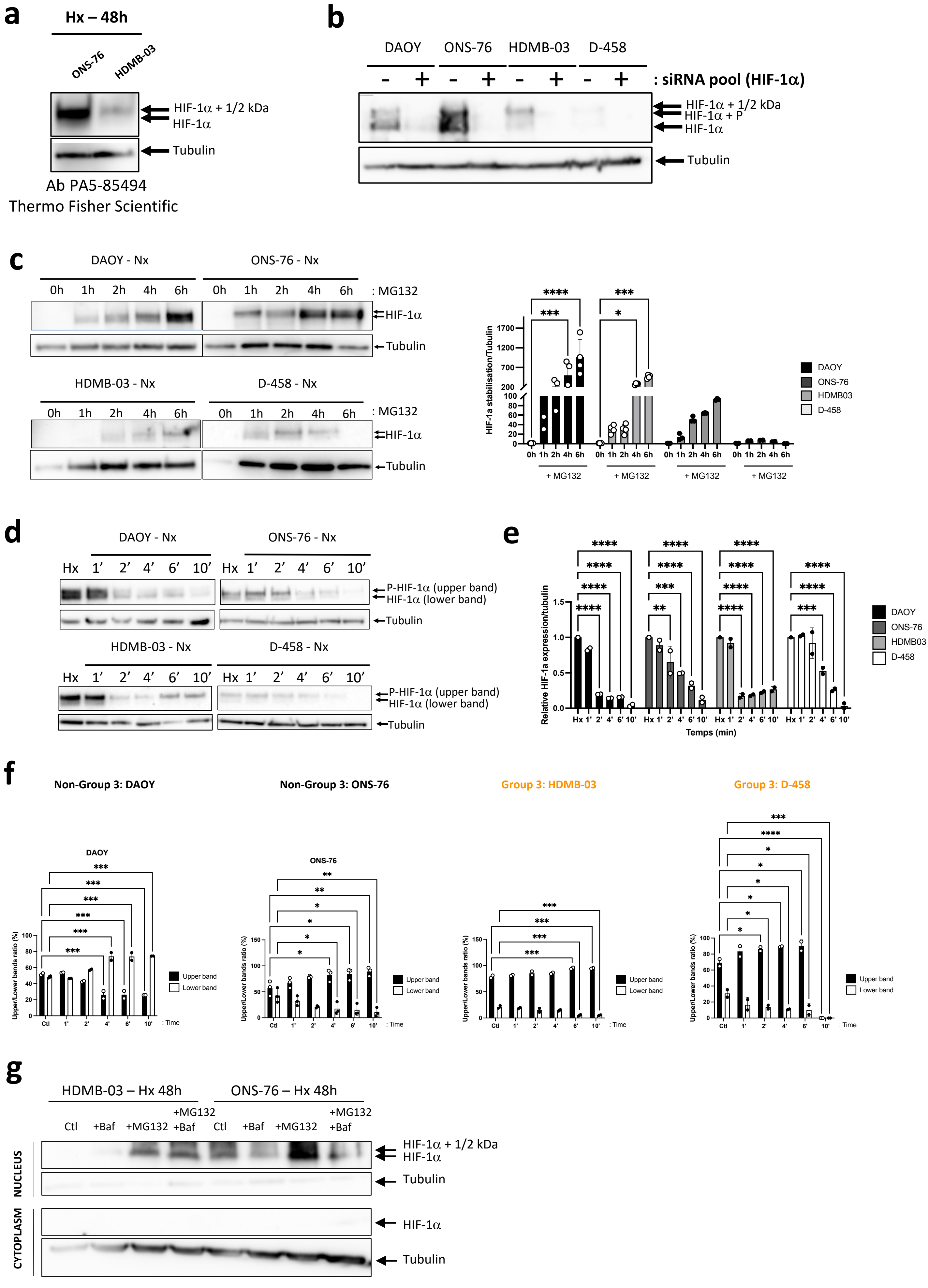

Suppl. FIGURE 4

a

- **Positions of Primers in HIF-1α cDNA**

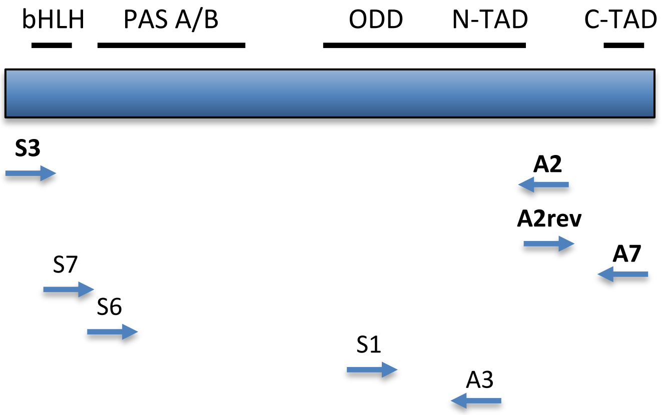

- **Primer sequences (5' to 3')**

S1 : CCCCAGATTCAGGATCAGACA
S3 : GTGAAGACATCGCGGGGAC
S6 : TGTGACCATGAGGAAATGAGAGA
S7 : GGATATTGAAGATGACATG
A2rev : GTTAACTGAGCTTTTTCTTA
A2 : TAAGAAAAAGCTCAGTTAAC
A3 : TCTAAATCTGTGTCCTCAGG
A7 : TCCACAGAAGATGTTTATTTG

b

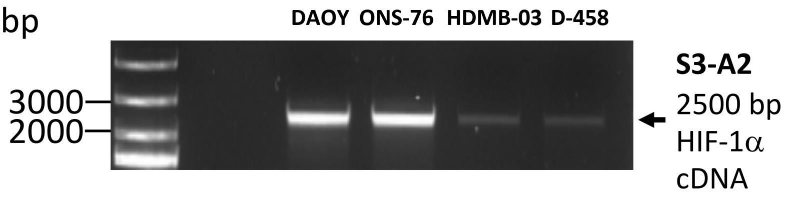

c

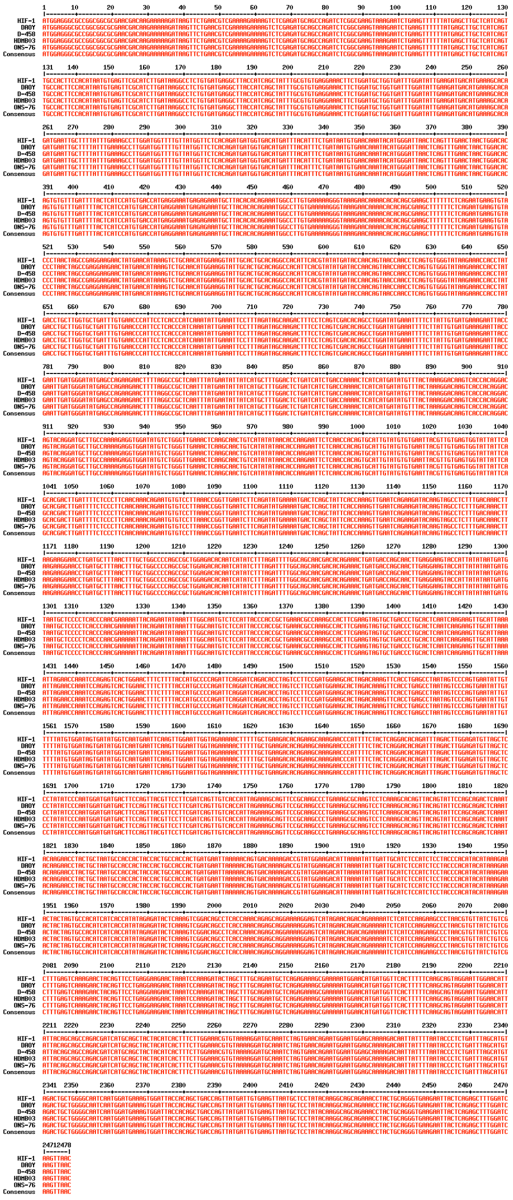

d

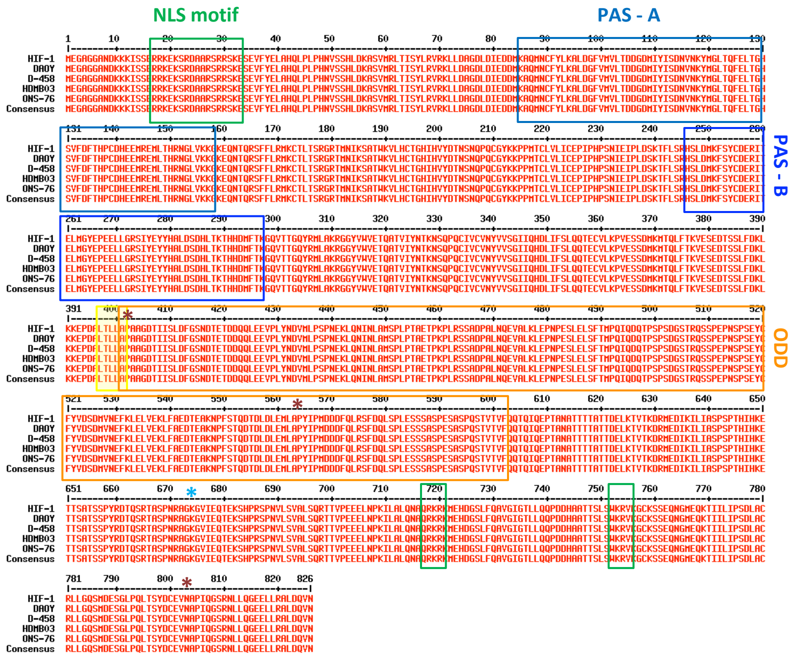

Suppl. FIGURE 5

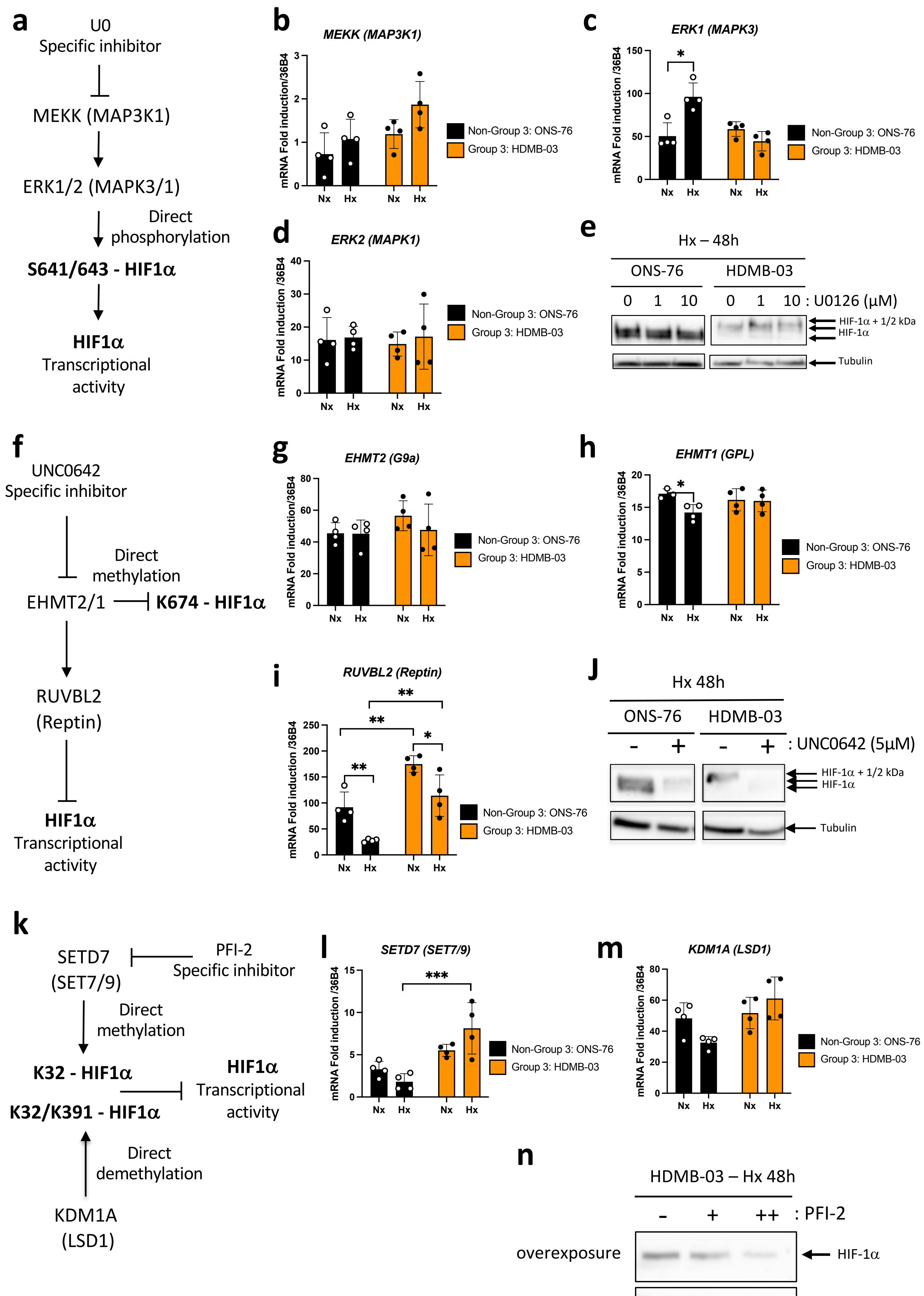

Suppl. FIGURE 6

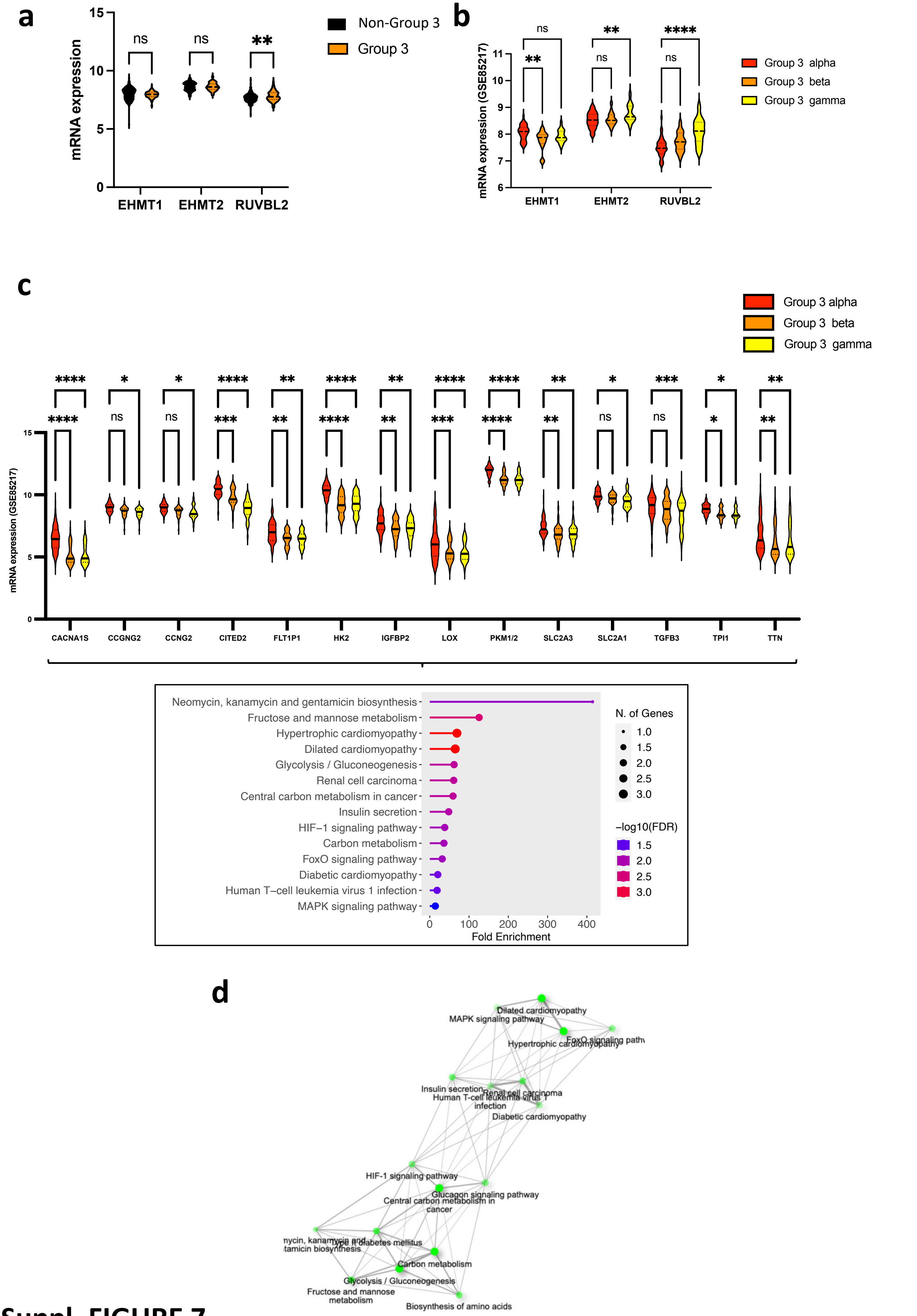

Suppl. FIGURE 7

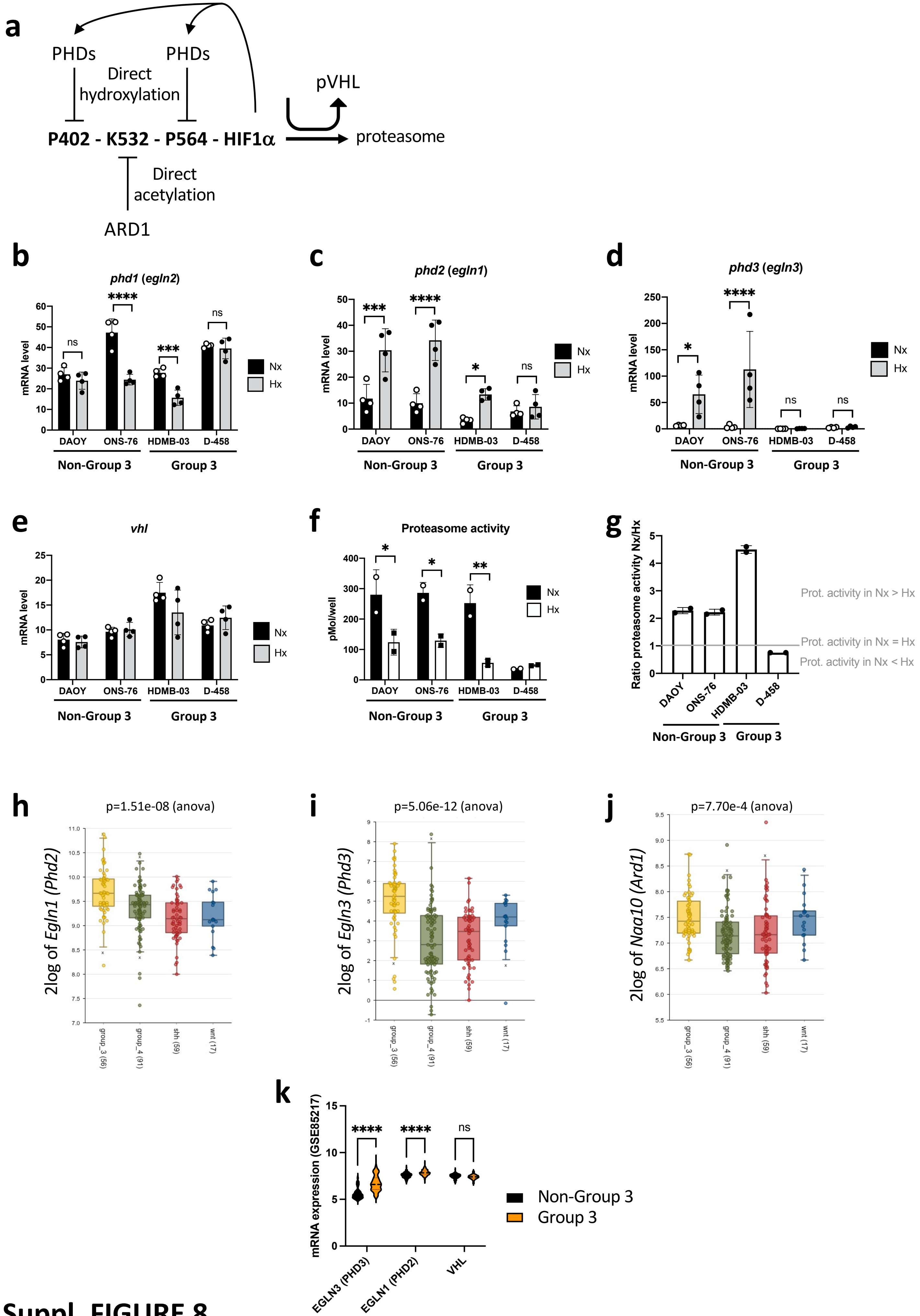

Suppl. FIGURE 8

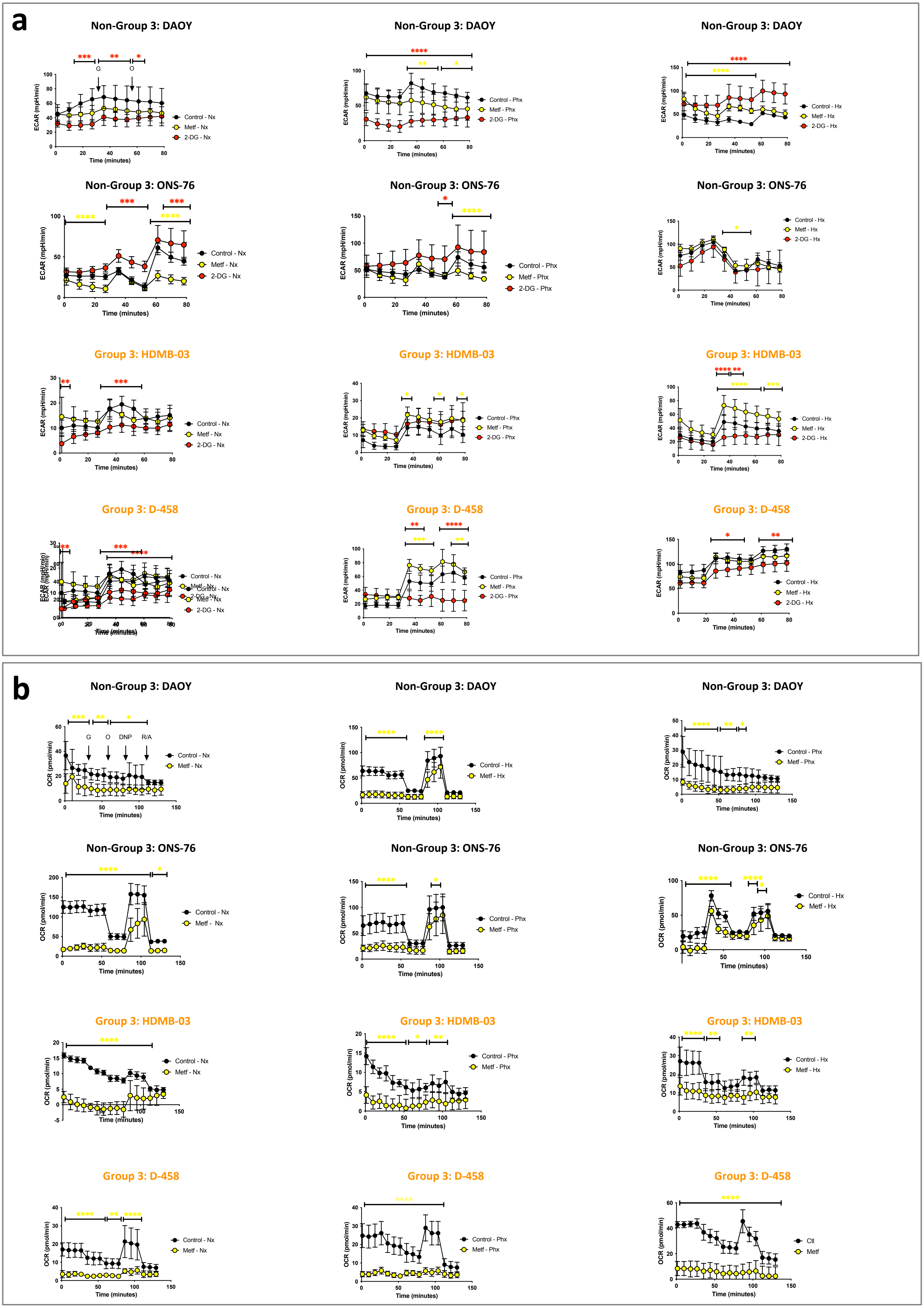

Suppl. FIGURE 9

a

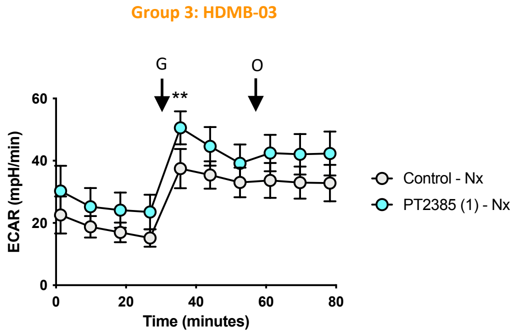

b

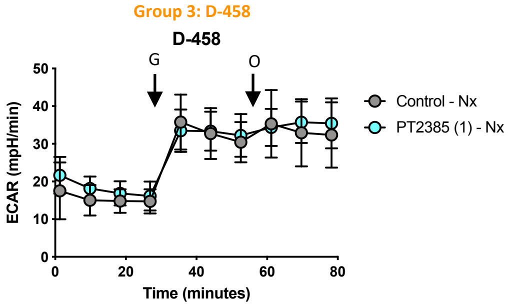

c

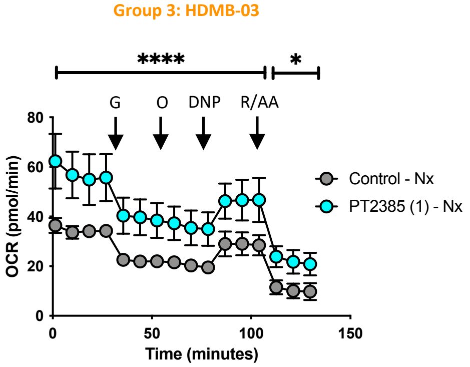

d

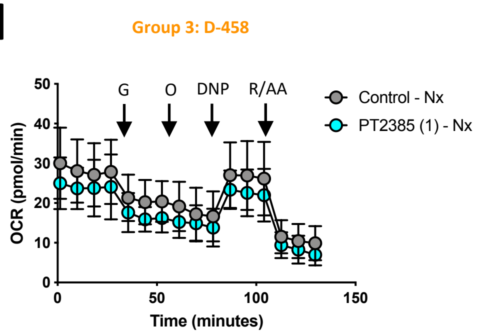

Suppl. FIGURE 10

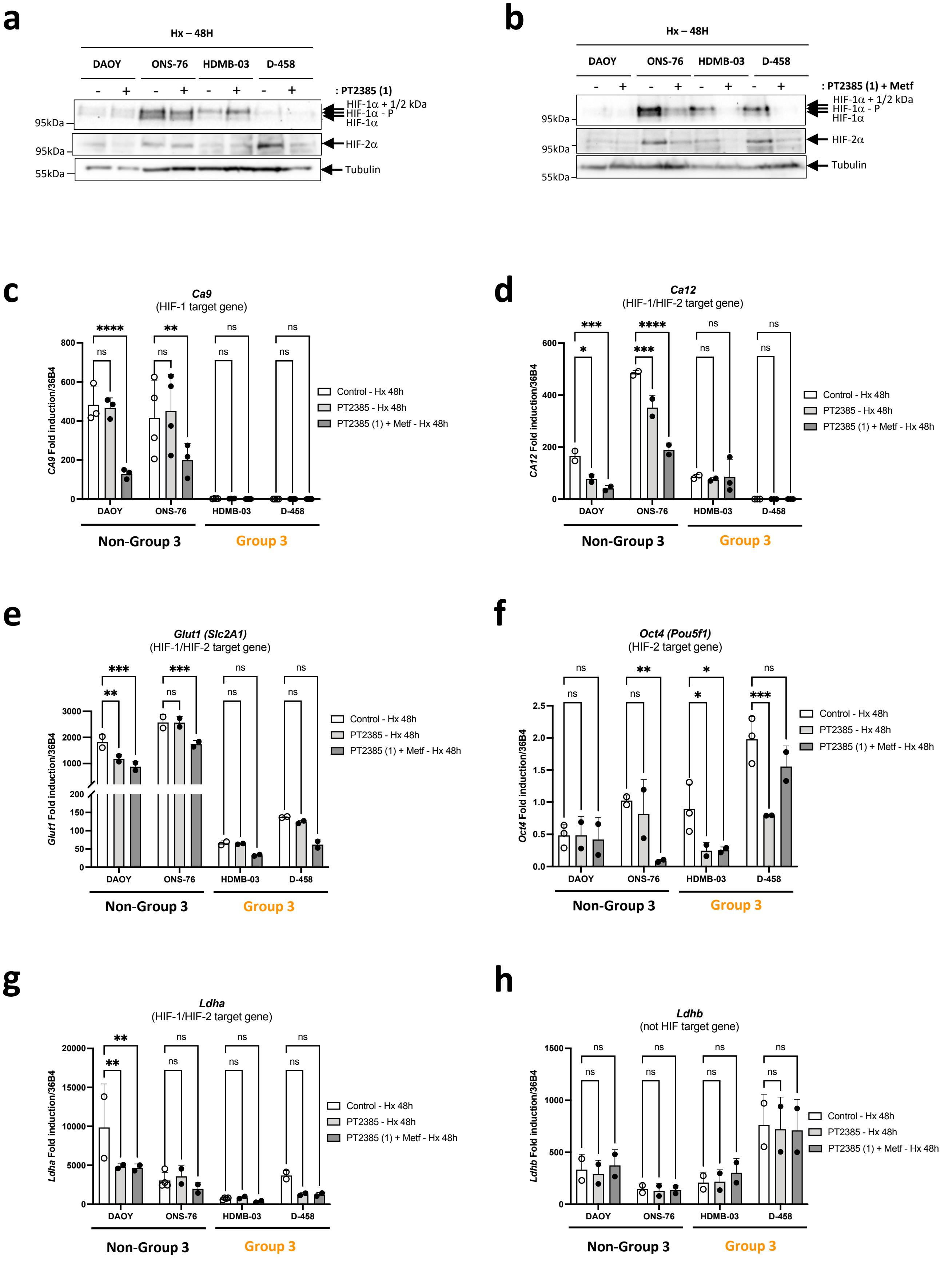

Suppl. FIGURE 11

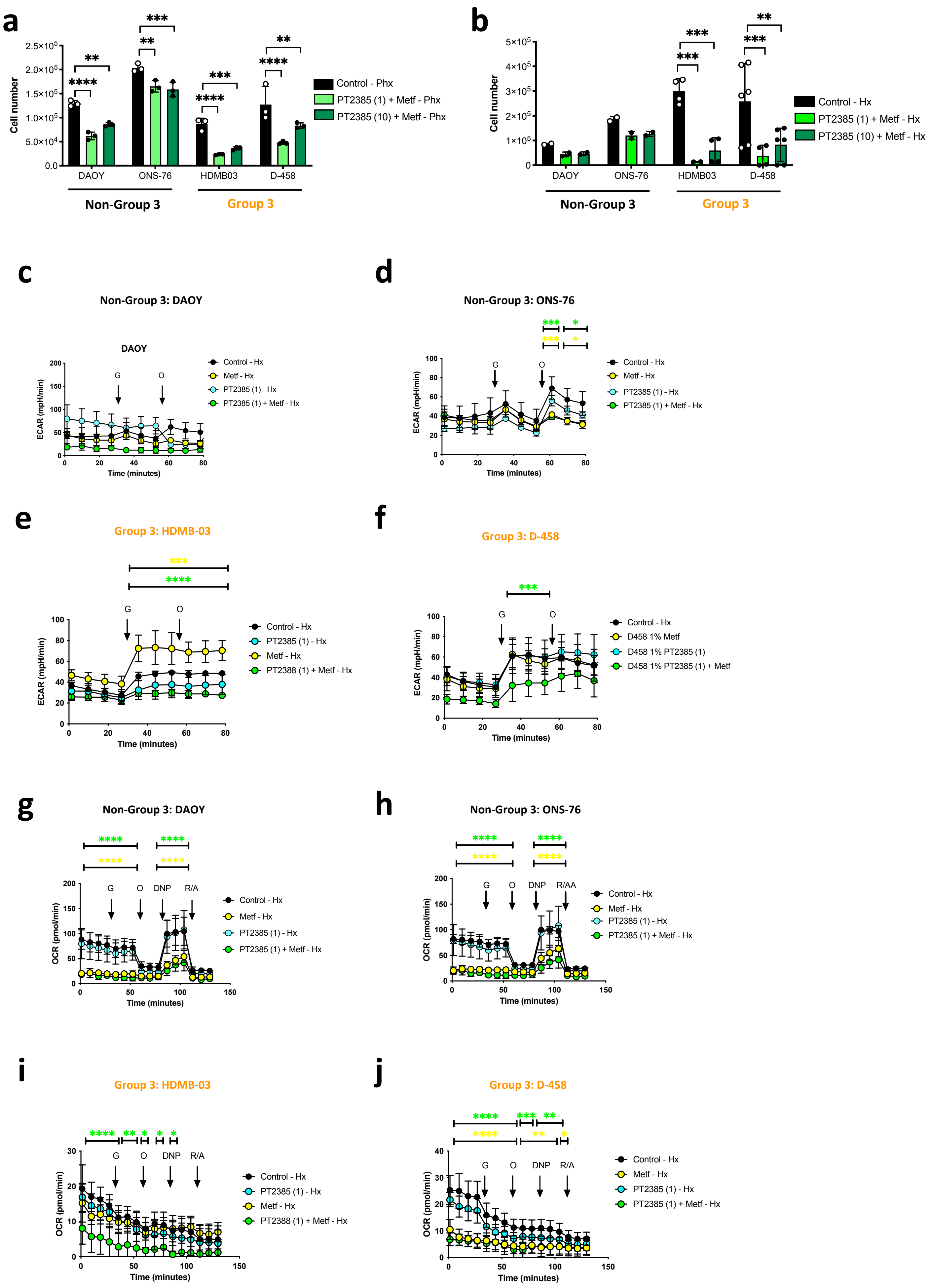

Suppl. FIGURE 12

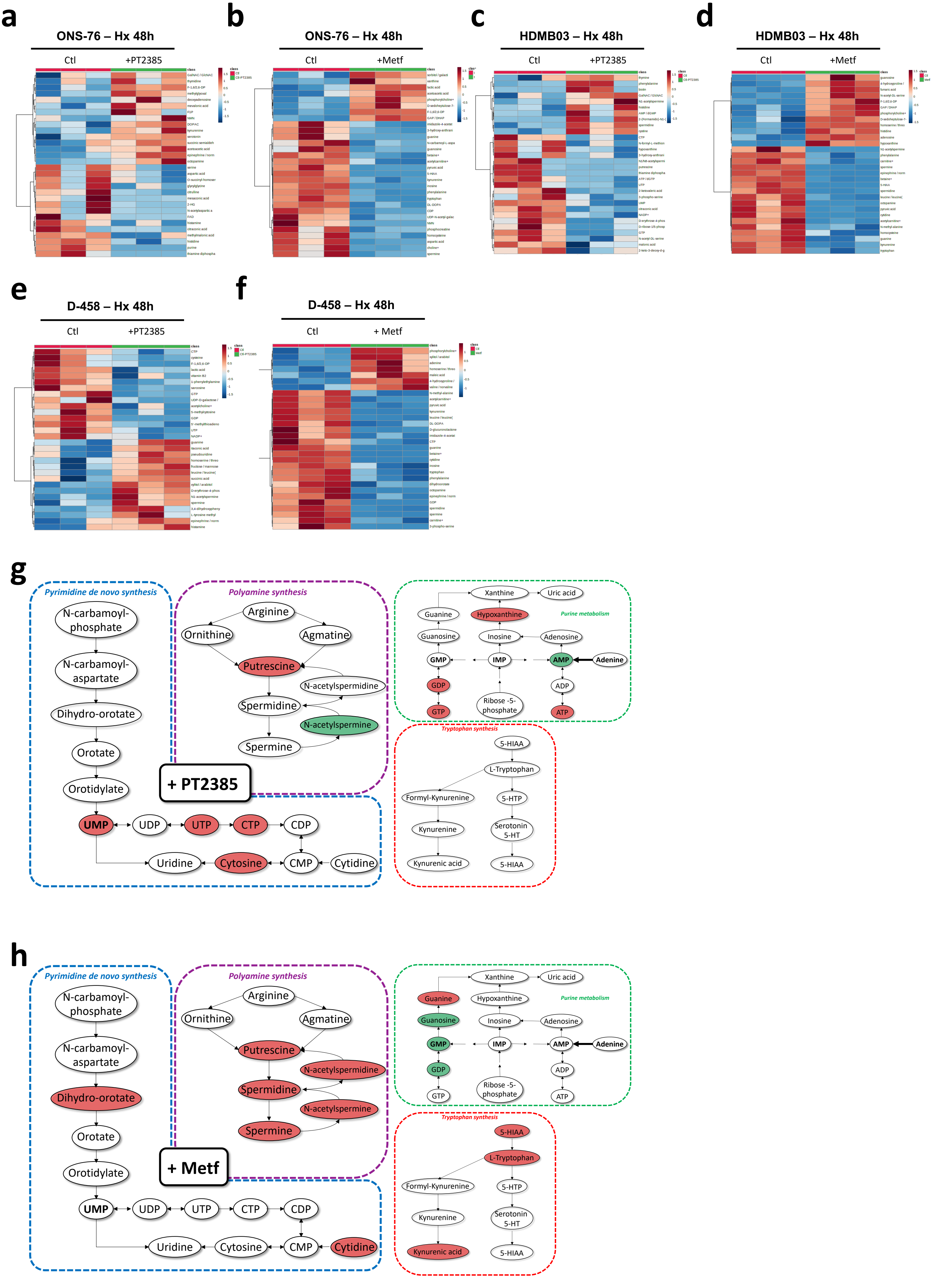

Suppl. FIGURE 13

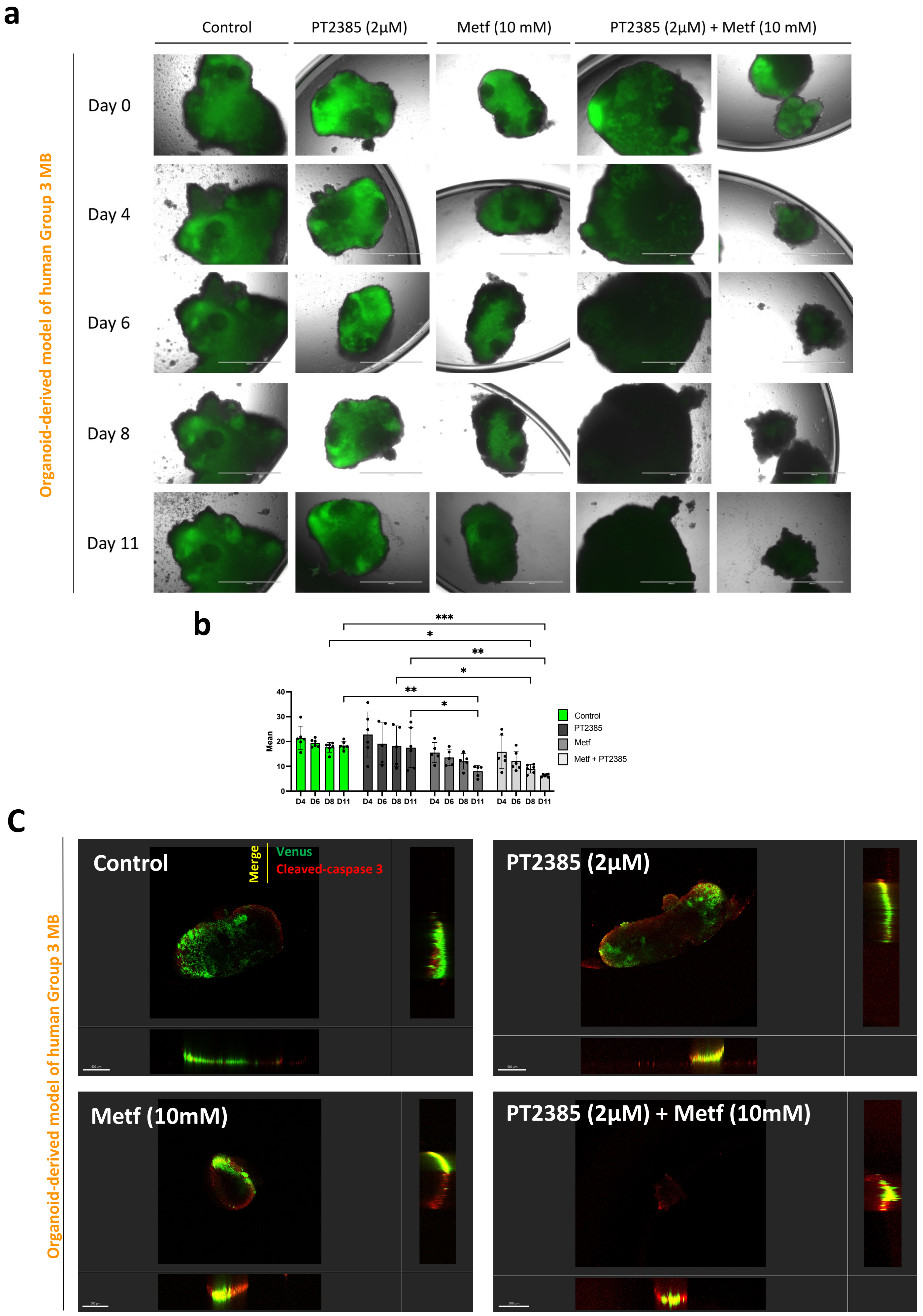

Suppl. FIGURE 14
