# Supplementary material for: HIF-1 inactivation empowers HIF-2 to drive hypoxia adaptation in aggressive forms of medulloblastoma
Source: Cell Death Discov. 2024 Jul 24;10:338. doi: 10.1038/s41420-024-02100-5 (PMC11269614; doi:10.1038/s41420-024-02100-5)
Supplement: Supplementary file 2 — Suppl. Legends [file 41420_2024_2100_MOESM2_ESM.pdf]

Suppl. Figure 1.

a-h: Graphic representation of (**a**) *Ca9*, (**b**) *Mct4*, (**c**) *Ca12*, (**d**) *Glut1*, (**e**) *Ldha*, (**f**) *Oct4*, (**g**) *Mct1* and (**h**) *Ldhb* mRNA expression in DAOY, ONS-76, HDMB-03, and D-458 cells incubated in Nx for 24h and Hx for 24, 48 and 72 h.

The 2-way ANOVA is representative of at least three independent experiments. * p<0.05, ** p<0.005, *** p<0.0005 and **** p<0.0001. **(i)** Heatmap of the RNA expression of genes in DAOY, ONS-76, HDMB-03 and D-458 cells exposed to Nx or Hx, compared with PDX3 and PDX7 from Group 3 MB.

Suppl. Figure 2.

a-h, Boxplot distributions of gene expressions of (**a**) *HIF-1α*, (**b**) *Angpt1*, (**c**) *Epo*, (**d**) *HIF-2α/EPAS*, (**e**) *Isl2*, (**f**) *Myc*, (**g**) *Vegfa* and (**h**) *Ldha* in Group 3, Group 4, SHH Group and WNT Group MB cells using the R2: Genomics analysis and visualization platform.

Suppl. Figure 3. (**a**) tSNE of 3725 cells colored by GSVA enrichment score for the Wnt subgroup gene signature: Group 3 (red), Group 4 (blue) and Wnt Group (green)[1].

b-j: Cells colored by classification for enrichment of Wnt subgroup signature. Dark red cells surpassed the 5% cutoff and are considered significantly enriched for the gene signature. Gray cells did not pass the threshold. (**b**) *Ca9*, (**c**) *Lox*, (**d**) *Glut1* (*Slc2a1*), (**e**) *Ldha*, (**f**) *Mpzl1*, (**g**) *Oct4* (*Pou5f1*), (**h**) *Icam4*, (**i**) *Mct1* (*Slc16a1*), (**j**) *ldhb* and (**k**) *Myc*.

(i) HIF-1, HIF-1/2 and HIF-2 percentage of expression in the different subgroups (WNT, GR4 and GR3).

Suppl. Figure 4.

(a) ONS-76 and HDMB-03 cell lysates were analyzed by immunoblotting for HIF-1α from Invitrogen (PA5-85494) and Tubulin was used as a loading control. (**b)** DAOY, ONS-76, HDMB-03 and D-458 cells were transfected with control siRNA (-) and a pool of siHIF-1α ((+) siHIF1pool). Cell lysates were analyzed by immunoblotting for HIF-1α, HIF-2α, and Tubulin was used as a loading control. **(c)** DAOY, ONS-76, HDMB-03 and D-458 cells were seeded at the same density and incubated in Nx (21% O_2) for 18h. MG132 (10µM) was added for 1, 2, 4 or 6 h. Cell lysates were analyzed by immunoblotting for HIF-1α. Tubulin was used as a loading control. Bottom panel, Quantification of HIF-1α protein levels. The 2-way ANOVA is representative of at least three independent experiments. * p<0.05, *** p<0.0005

and **** $p<0.0001$. (**d)** DAOY, ONS-76, HDMB-03, and D-458 cells were incubated in hypoxia (Hx) for 48h and reoxygenated for 1, 2, 4, 6, and 10 min ('). Cell lysates were analyzed by immunoblotting for HIF-1α. Tubulin was used as a loading control. (**e)** Quantitative analysis of the total HIF-1α bands compared to tubulin. (**f)** Quantitative analysis of the upper and lower HIF-1α bands compared to tubulin. (**g)** HDM-03 and ONS-76 cells were seeded at the same density and incubated in Hx (1% O_2) for 48h. Bafilomycin (Baf) or MG132 or both (+MG132+Baf) were added 6h prior to lysis. Subcellular fractionation was used to identify proteins in nuclei and cytoplasm. Tubulin was used as a loading control.

Suppl. Figure 5. (a) Summary of primers used for RT-PCR. (**b**) Representative experiments of RT-PCR of HIF-1α cDNA. (**c**) Alignment of the cDNA of HIF-1α (NCBI Reference Sequence: NC_000014.9) with the cDNA of HIF-1α from DAOY, ONS-76, HDMAB03 and D-458 cells. (**d**) Alignment of the HIF-1α protein sequences from HIF-1α control (sp|Q16665|HIF1A_HUMAN Hypoxia-inducible factor 1-alpha), DAOY, ONS-76, HDMB-03 and D-458.

Suppl. Figure 6. (a) Molecular signal affecting the phosphorylation of HIF-1α.
b-d: Graphic representation of (**b**) *Mekk (MAP3K1),* (**c**) *Erk1 (Mapk3),* and (**d**) *Erk2 (Mapk1)* mRNA expression in ONS-76 and HDMB-03 cells incubated in normoxia (Nx) or hypoxia (Hx - 1% O_2) for 24h. The 2-way ANOVA is representative of at least three independent experiments. * $p<0.05$. **e**, ONS-76 and HDMB-03 cells were seeded at the same density and incubated in Hx (1% O_2) for 48h in the absence (-) or presence of (1 and 10 µM) U0126. ONS-76 and HDMB-03 cell lysates were analyzed by immunoblotting for HIF-1α and Tubulin was used as a loading control. **(f)** Molecular signal affecting the methylation of HIF-1α through EHMT1/2. **g-i:** Graphic representation of (**g**) *Ehmt2 (G9a),* (**h**) *Ehmt1 (Gpl),* and (**i**) *Ruvbl2 (Reptin)* mRNA expression in ONS-76 and HDMB-03 cells incubated in Nx or Hx for 24h. The 2-way ANOVA is representative of at least three independent experiments. * $p<0.05$ and ** $p<0.005$. (**j)** ONS-76 and HDMB-03 cells were seeded at the same density and incubated in Hx for 48h in the absence (-) or presence (+) of UNC0642 (5µM). ONS-76 and HDMB-03 cell lysates were analyzed by immunoblotting for HIF-1α and Tubulin was used as a loading control. **(k)** Molecular signal affecting the phosphorylation of HIF-1α through SETD7. **l-m:** Graphic representation of (**l**) *Setd7 (Set7/9)* and (**m**) *Kdm1a (Lsd1)* mRNA expression in ONS-76 and HDMB-03 cells incubated in Nx or Hx for 24h. The 2-way ANOVA is representative

of at least three independent experiments. *** $p<0.0005$. (**n)** ONS-76 and HDMB-03 cells were seeded at the same density and incubated in Hx (1% O_2) for 48h in the absence (-) or presence (+) of PFI-2 (2 nM (+) and 2µM (++)). ONS-76 and HDMB-03 cell lysates were analyzed by immunoblotting for HIF-1α and Tubulin was used as a loading control.

Suppl. Figure 7. a-b: Violin distributions of gene expressions of *Ehmt1, Ehmt2* and *Ruvbl2* in (**a**) Non-Group 3 *versus* Group 3 MB and (**b**) subgroups (alpha, beta and gamma) of Group 3 MB. (**c**) Top - Violin distributions of HIF-1 target gene expressions in subgroups (alpha, beta and gamma) of Group 3. Bottom - KEGG Fold enrichment from ShinyGo 0.77. **(a-c)** The 2-way ANOVA is representative of at least three independent experiments. * $p<0.05$, ** $p<0.005$, *** $p<0.0005$ and **** $p<0.0001$. (**d**) Network representation of KEGG from ShinyGo 0.77.

Suppl. Figure 8. (a) Molecular signal affecting the hydroxylation and acetylation of HIF-1α. **b-e**, Graphic representation of (**b**) *Phd1 (Egln2)*, (**c**) *Phd2 (Egln1)*, (**d**) *Phd3 (Egln3)* and (**e**) *Vhl* mRNA expression in ONS-76 and HDMB-03 cells incubated in normoxia (Nx) for 24h or hypoxia (Hx - 1% O_2) for 48h. The 2-way ANOVA is representative of at least three independent experiments. * $p<0.05$, *** $p<0.0005$ and **** $p<0.0001$. **f-g:** DAOY, ONS-76, HDMB-03 and D-458 cells were seeded at the same density and incubated in Nx or Hx for 48h. (**f**) Proteasome activity in the cells, measured in pMol/well. The 2-way ANOVA is representative of two independent experiments. * $p<0.05$ and ** $p<0.005$. (**g**) Graph showing the ratio of the proteasome activity obtained in Nx *versus* Hx for each of the cells. **h-j:** Boxplot distributions of gene expressions of (**h**) *egln1* (*phd2*), (**i**) *egln3* (*phd3*) and (**j**) *naa10* (*ard1*). (**k**) Violin distributions of gene expressions of *Egln3 (Phd3), Egln1 (Phd2)* and *vhl* in Non-Group 3 *versus* Group 3 MB.

Suppl. Figure 9. (a) ECAR of DAOY (first line), ONS-76 (second line), HDMB-03 (third line) and D-458 (fourth line) cells cultured in Nx (21% O_2 – first column), Phx (6% O_2 – second column) and Hx (1% O_2 – third column) in the presence of Metformin (Metf) or 2-DG for 24 h was evaluated with the XF24 analyzer. Cells were deprived of glucose for 1 h, then glucose (G) and oligomycin (O) were injected. The graphs are representative of at least three independent experiments carried out in octuplicate. Yellow star (*) represents the statistical differences between Metf and control and red (*) star between 2-DG and control. The 2-way ANOVA is

representative of at least three independent experiments. * $p<0.05$, ** $p<0.005$, *** $p< 0.001$ and ****$p<0.0001$.

(**b**) OCR of DAOY (first line), ONS-76 (second line), HDMB-03 (third line) and D-458 (fourth line) cells cultured in Nx (21% O_2 – first column), Phx (6% O_2 – second column) and Hx (1% O_2 – third column) in the presence of Metformin (Metf) or 2-DG for 24 h was evaluated with the XF24 analyzer. Cells were deprived of glucose for 1h, then glucose (G), oligomycin (O), DNP, and Rotenone + Antimycin A (R/A) were injected at the indicated times. The graphs are representative of at least three independent experiments carried out in octuplicate. Yellow star (*) represents the statistical differences between Metf and control. The 2-way ANOVA is representative of at least three independent experiments. * $p<0.05$, ** $p<0.005$, *** $p< 0.001$ and ****$p<0.0001$.

Suppl. Figure 10. a-b, ECAR of (**a**) HDMB-03 and (**b**) D-458 cells in Nx in the absence (Control) or presence of PT2385 (1µM) for 24h was evaluated with the XF24 analyzer. Cells were deprived of glucose for 1h, then glucose (G) and oligomycin (O) were injected at the indicated times. The graphs are representative of at least three independent experiments carried out in octuplicate. **c-d,** Respiratory control of **(c)** HDMB-03 and **(d)** D-458 cells. OCR was measured in real time with the XF24 analyzer. Cells were cultured for 24h in Nx in the absence (Control) or presence of PT2385 (1µM). Cells were deprived of glucose for 1h, then glucose (G), oligomycin (O), DNP, and Rotenone + Antimycin A (R/A) were injected at the indicated times. The graphs are representative of at least three independent experiments carried out in octuplicate. * $p<0.05$, ** $p<0.005$ and ****$p<0.0001$.

Suppl. Figure 11. (a) DAOY, ONS-76, HDMB-03 and D-458 cells were incubated in hypoxia (Hx - 1% O_2) for 72 h in the absence (-) or presence (+) of PT2385 (1µM). Cell lysates were analyzed by immunoblotting for HIF-1α and HIF-2α. Tubulin was used as a loading control. (**b**) DAOY, ONS-76, HDMB-03 and D-458 cells were incubated in Hx for 72 h in the absence (-) or presence (+) of PT2385 (1µM) and Metformin (Metf – 10mM). Cell lysates were analyzed by immunoblotting for HIF-1α and HIF-2α. Tubulin was used as a loading control. **c-h:** Graphic representation of (**c**) *Ca9*, (**d**) *Ca12*, (**e**) *Glut1 (Slc2A1)*, (**f**) *Oct4 (Pou5f1)*, (**g**) *Ldha* and (**h**) *Ldhb* mRNA expression in DAOY, ONS-76, HDMB-03 and D-458 cells incubated in Hx for 48h in the absence (Control) or presence of PT2385 (1µM) or the presence of both PT2385 (1µM) and Metformin (Metf – 10mM). The 2-way ANOVA is representative of at least

three independent experiments. Non-significant (ns), * p<0.05, ** p<0.005, *** p< 0.001 and ****p<0.0001.

Suppl. Figure 12. a-b, DAOY, ONS-76, HDMB-03 and D-458 cells were incubated in **(a)** physioxia (Phx) and **(b)** hypoxia (Hx – **b**) for 72h in the absence (Control) or presence of PT2385 (1µM or 10µM) and Metformin (Metf – 10mM). Cell number was measured using an ADAM cell counter. The 2-way ANOVA is representative of at least three independent experiments. ** p<0.005, *** p< 0.001 and ****p<0.0001. **c-f:** ECAR of (**c**) DAOY, (**d**) ONS-76, (**e**) HDMB-03 and (**f**) D-458 cells cultured in Hx in the presence of Metformin (Metf – 10mM), PT2385 (1µM) or both for 24h was evaluated with the XF24 analyzer. Cells were deprived of glucose for 1h, then glucose (G) and oligomycin (O) were injected. Yellow star (*) represents the statistical differences between Metf and control and green (*) star between PT2385+Metf and control. The 2-way ANOVA is representative of at least three independent experiments. * p<0.05, *** p< 0.001 and ****p<0.0001. **g-j:** OCR of (**g**) DAOY, (**h**) ONS-76, (**i**) HDMB-03 and (**j**) D-458 cells cultured in Hx in the presence of Metformin (Metf – 10 mM), PT2385 (1µM) or both for 24 h was evaluated with the XF24 analyzer. Cells were deprived of glucose for 1h, then glucose (G), oligomycin (O), DNP, and Rotenone + Antimycin A (R/A) were injected at the indicated times. Yellow star (*) represents the statistical differences between Metf and control and green (*) star between PT2385+Metf and control. The 2-way ANOVA is representative of at least three independent experiments. * p<0.05, ** p<0.005, *** p< 0.001 and ****p<0.0001.

Suppl. Figure 13. a-f, Steady-state metabolite profile of (**a-b**) ONS-76, (**c-d**) HDMB-03 and (**e-f**) D-458 cells submitted to hypoxia (Hx) for 48h in the absence (Ctl), or presence of PT2385 or Metf. Intracellular metabolites from three independent samples per condition were profiled by LC/MS-MS, and those significantly altered in treated cells, relative to control cells, are shown as row-normalized heatmaps ranked according to fold-change $\log_2$ (treated/untreated). **(g)** Schematic representation of the different major metabolic pathways impacted by PT2385 treatment in Group 3 MB *versus* ONS-76. **h,** Schematic representation of the different major metabolic pathways impacted by Metf treatment in Group 3 MB *versus* ONS-76.

Suppl. Figure 14. (a) Validation of drug response in human Group 3 MB tumor organoids. Brightfield and fluorescence images of cerebellar organoids at day 0-, day 4-, day 6-, day 8-, day 11- and day 14- of treatment, electroporated with pBase + pPBMYC + pPBOtx2 +

pPBVenus. **(b)** Quantification of the mean (raw integrated density/area using Fiji) for each condition from day 4 (D4) to day 14 (D14). The ordinary two-way ANOVA is representative of at least three independent organoids. * $p<0.05$, ** $p<0.005$ and *** $p=0.0006$. **(c)** Superimposition images where the presence of both fluors, Venus (tumor cells) and red (cleaved- caspase 3) is shown as a third color (yellow - merge). Human Group 3 MB tumor organoids have been treated in the absence (Ctl) or presence of PT2385, Metf or the combination. The orthogonal view is used to show virtual cross sections – one plotted along the x-axis and the other plotted along the y-axis; scale bars = 100 µm.

1 Manoranjan, B., Adile, A. A., Venugopal, C. & Singh, S. K. WNT: an unexpected tumor suppressor in medulloblastoma. *Mol Cell Oncol* **7**, 1834903 (2020). https://doi.org:10.1080/23723556.2020.1834903
