# Supplementary material for: HIF-1 inactivation empowers HIF-2 to drive hypoxia adaptation in aggressive forms of medulloblastoma
Source: Cell Death Discov. 2024 Jul 24;10:338. doi: 10.1038/s41420-024-02100-5 (PMC11269614; doi:10.1038/s41420-024-02100-5)

Figure 1I

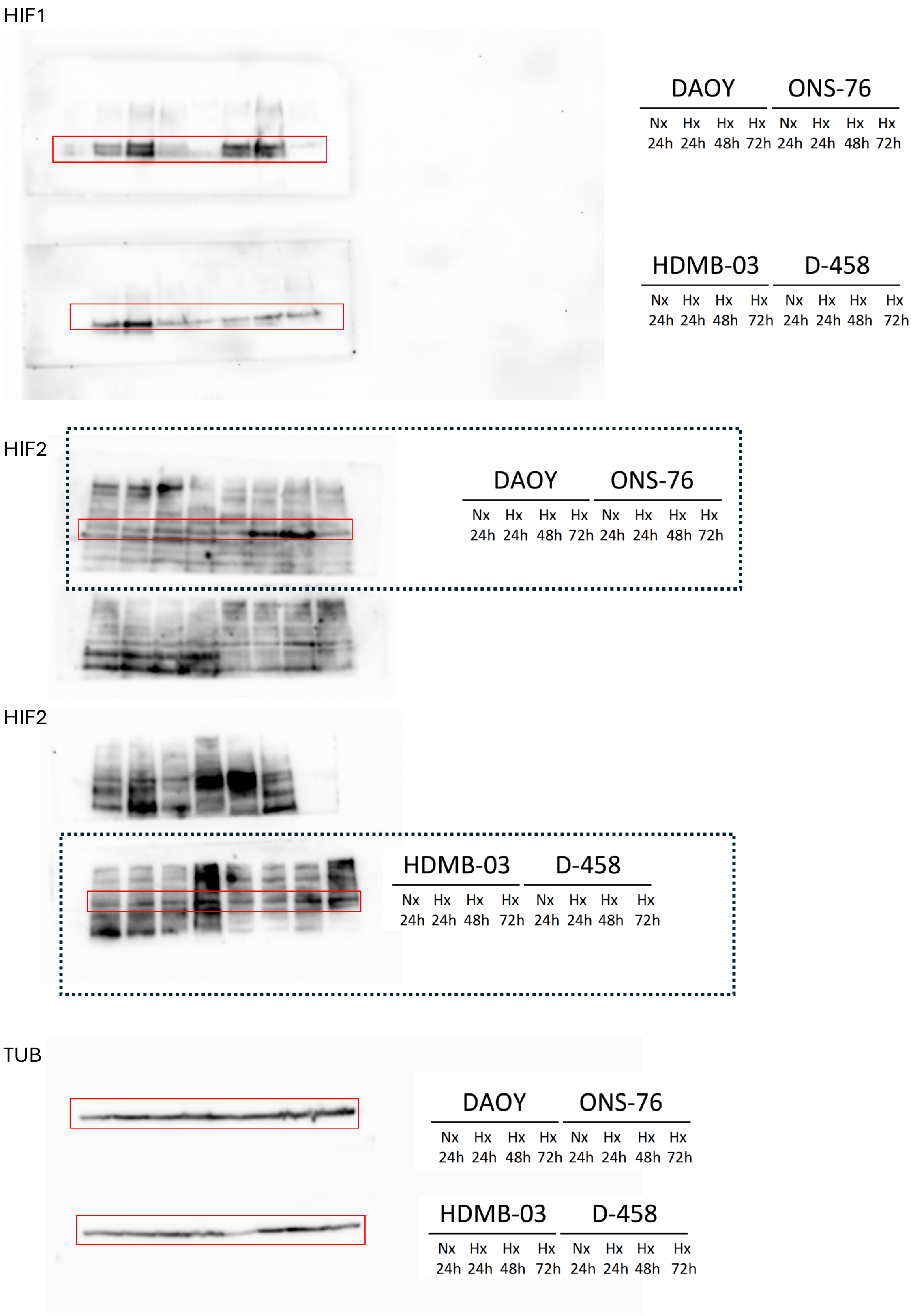
HIF1
DAOY
ONS-76
Nx 24h Hx 24h Hx 48h Hx 72h Nx 24h Hx 24h Hx 48h Hx 72h
HDMB-03
D-458
Nx 24h Hx 24h Hx 48h Hx 72h Nx 24h Hx 24h Hx 48h Hx 72h
HIF2
DAOY
ONS-76
Nx 24h Hx 24h Hx 48h Hx 72h Nx 24h Hx 24h Hx 48h Hx 72h
HIF2
HDMB-03
D-458
Nx 24h Hx 24h Hx 48h Hx 72h Nx 24h Hx 24h Hx 48h Hx 72h
TUB
DAOY
ONS-76
Nx 24h Hx 24h Hx 48h Hx 72h Nx 24h Hx 24h Hx 48h Hx 72h
HDMB-03
D-458
Nx 24h Hx 24h Hx 48h Hx 72h Nx 24h Hx 24h Hx 48h Hx 72h

Figure 2A

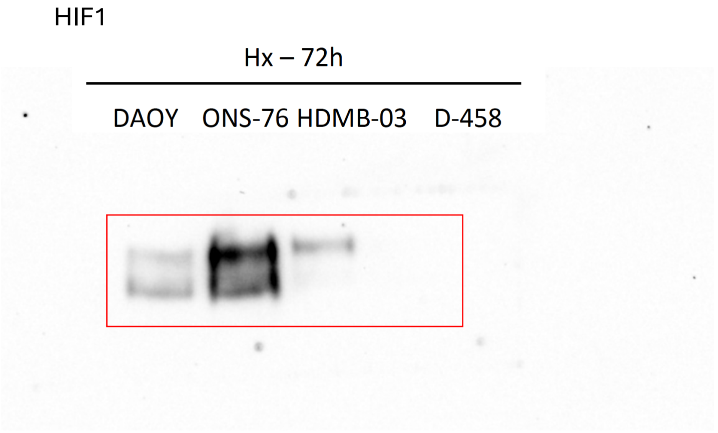

Figure 2E

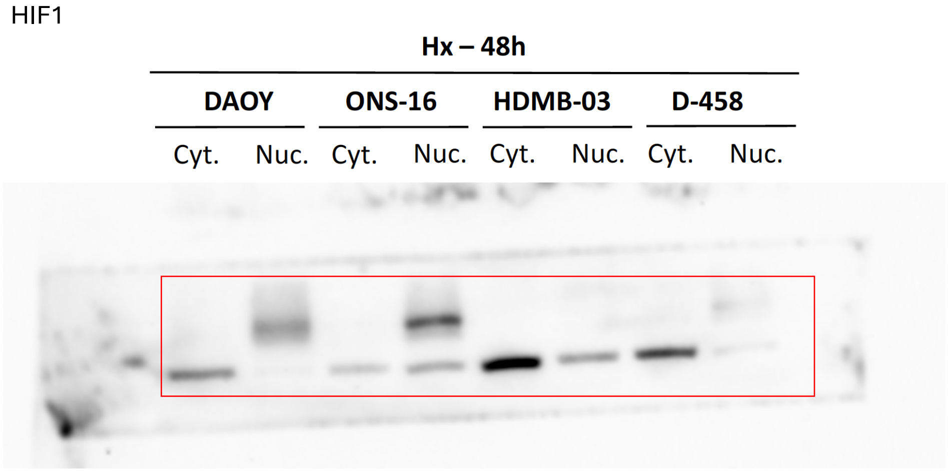

Suppl. Figure 4 a

HIF1

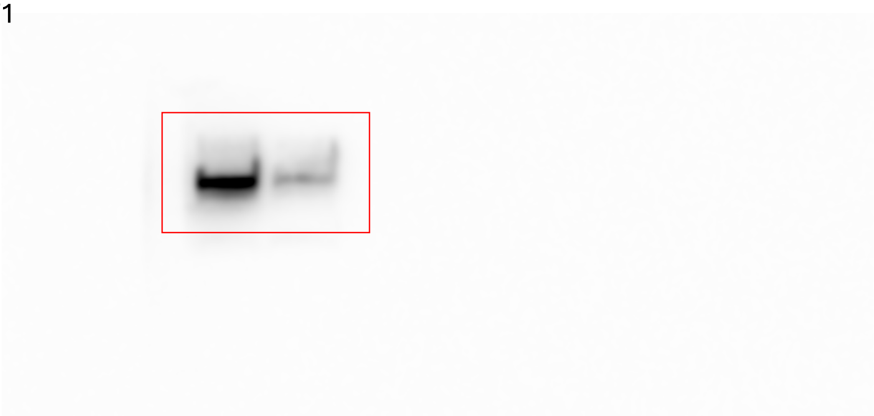

TUB

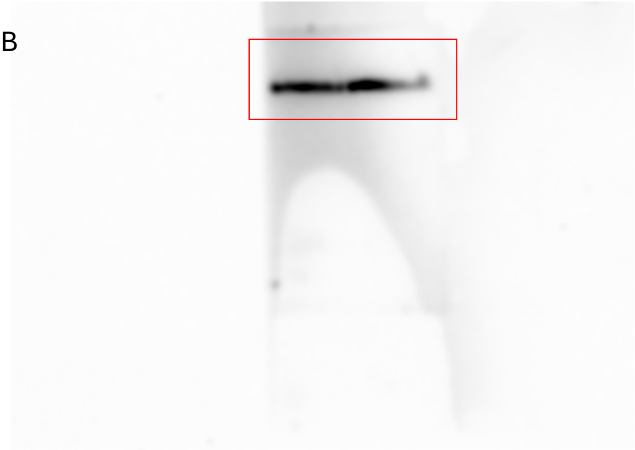

Suppl. Figure 4 b

HIF

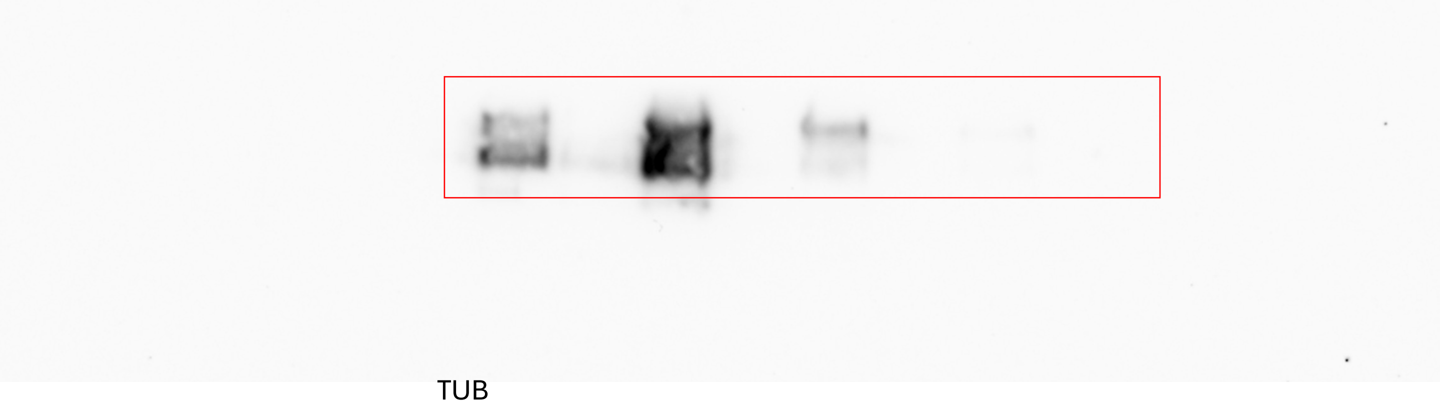

TUB

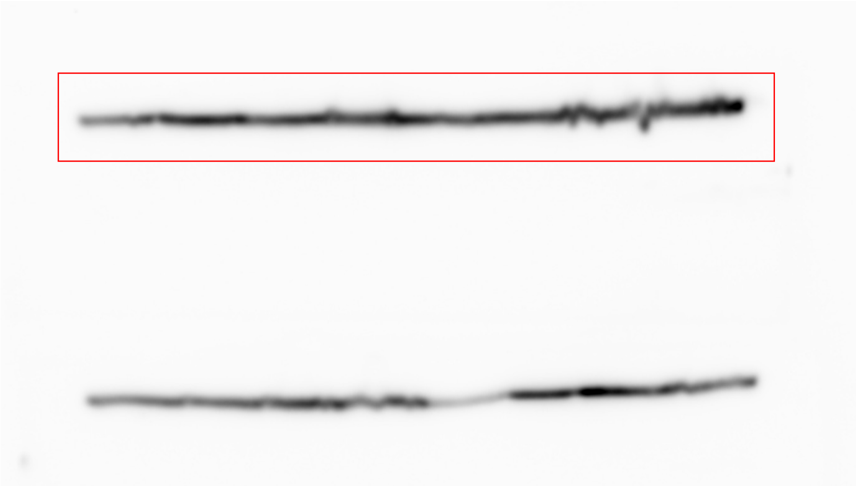

Suppl. Figure 4 C

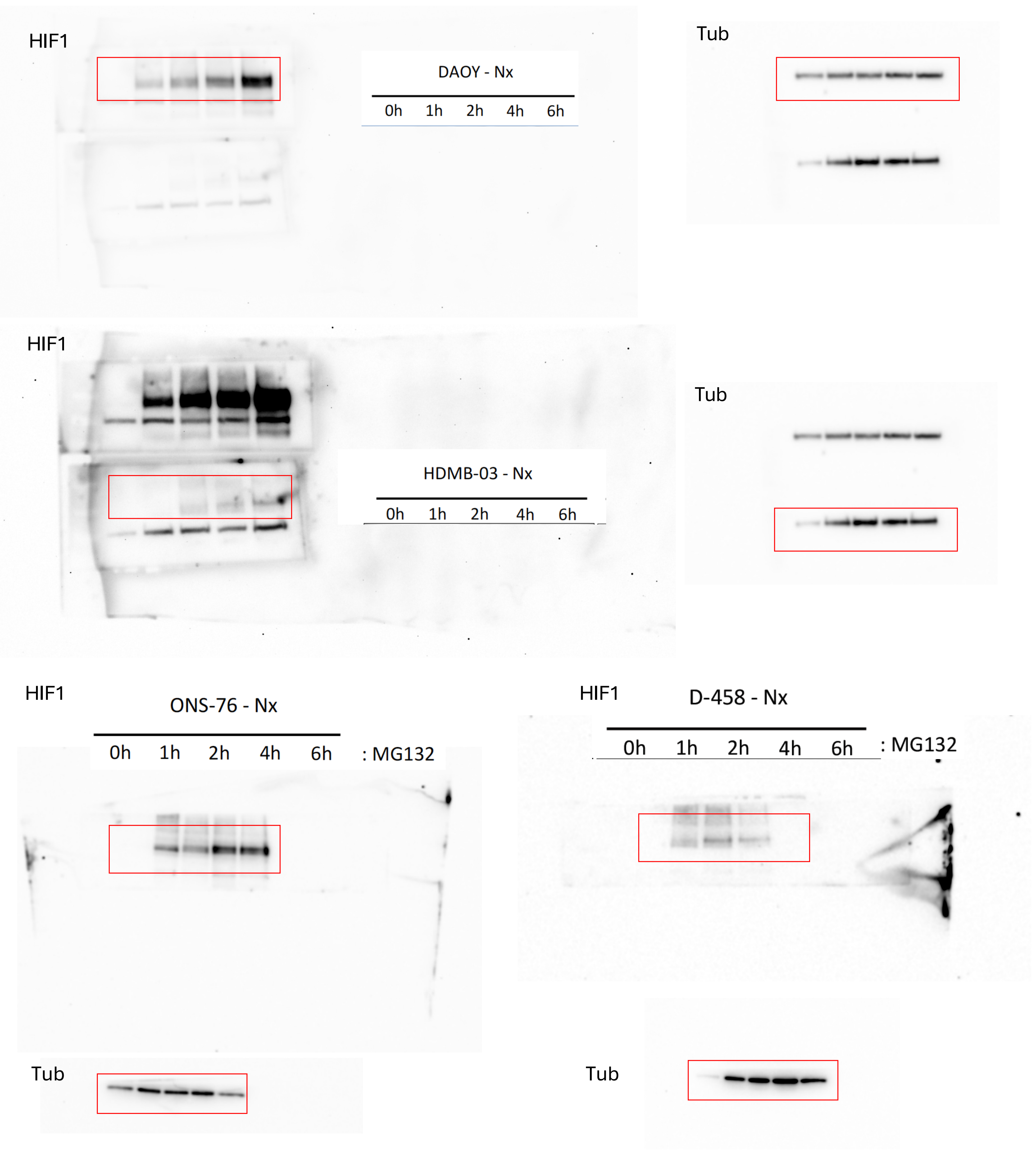

S Figure 4 d

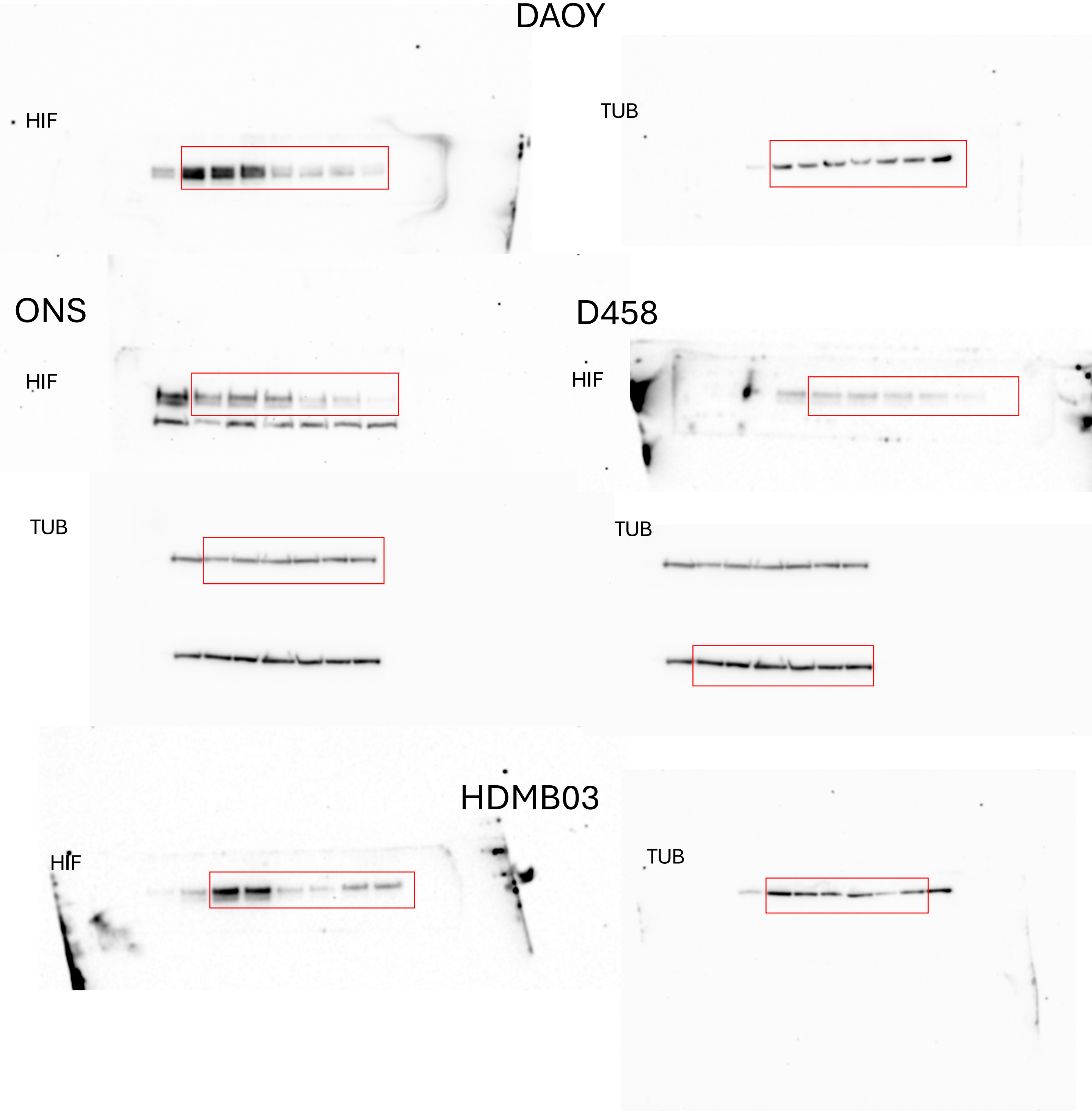

Suppl. Figure 4 g

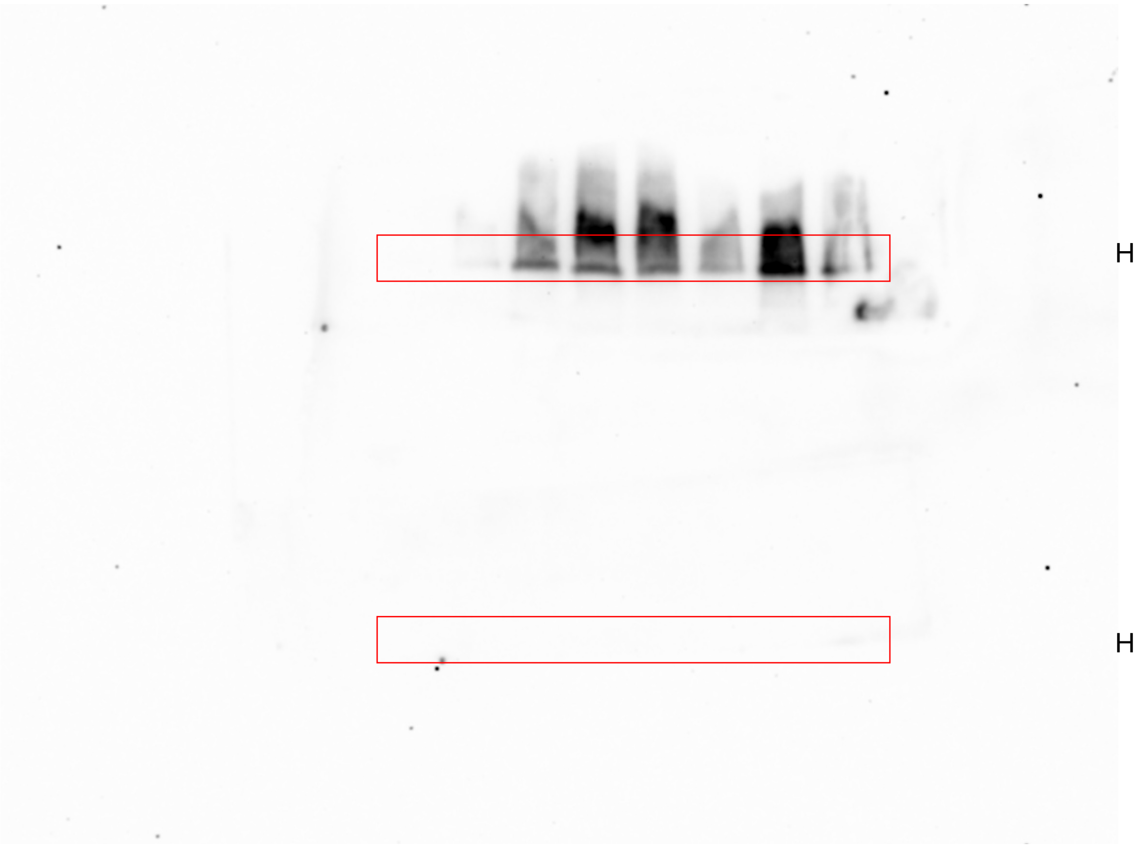

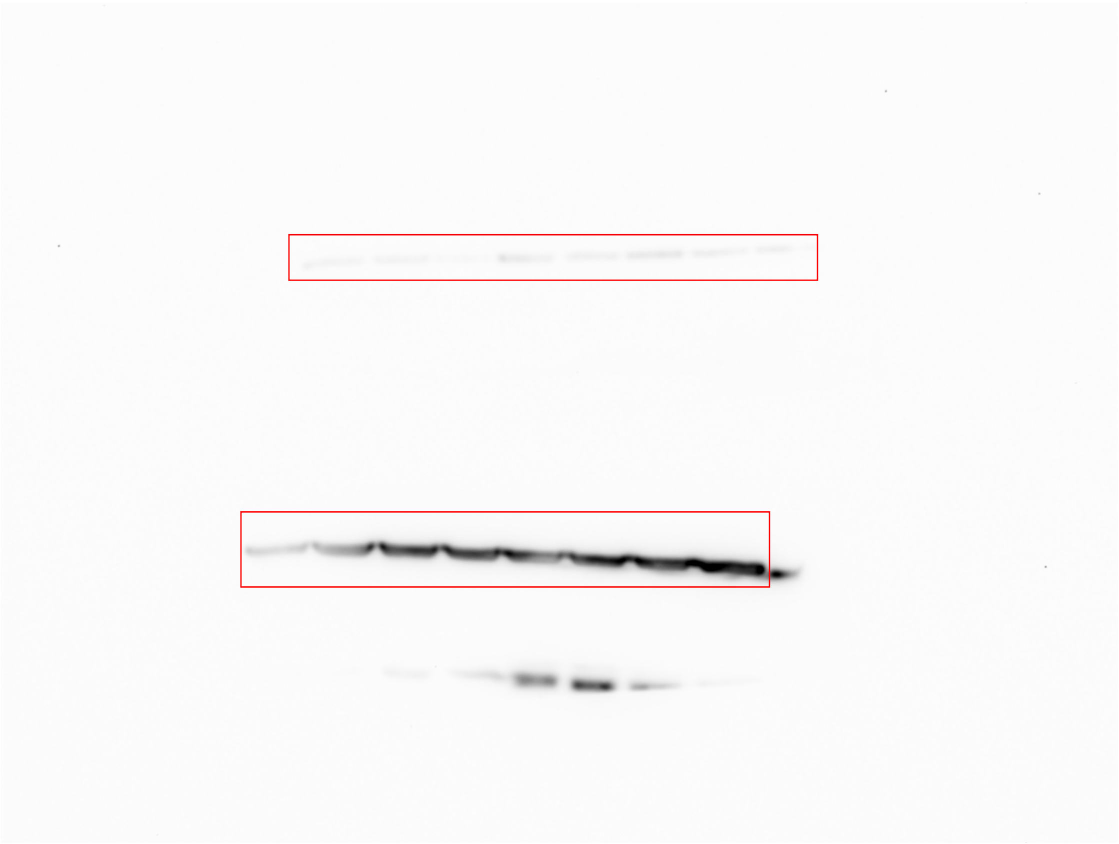

Suppl. Figure 6 e

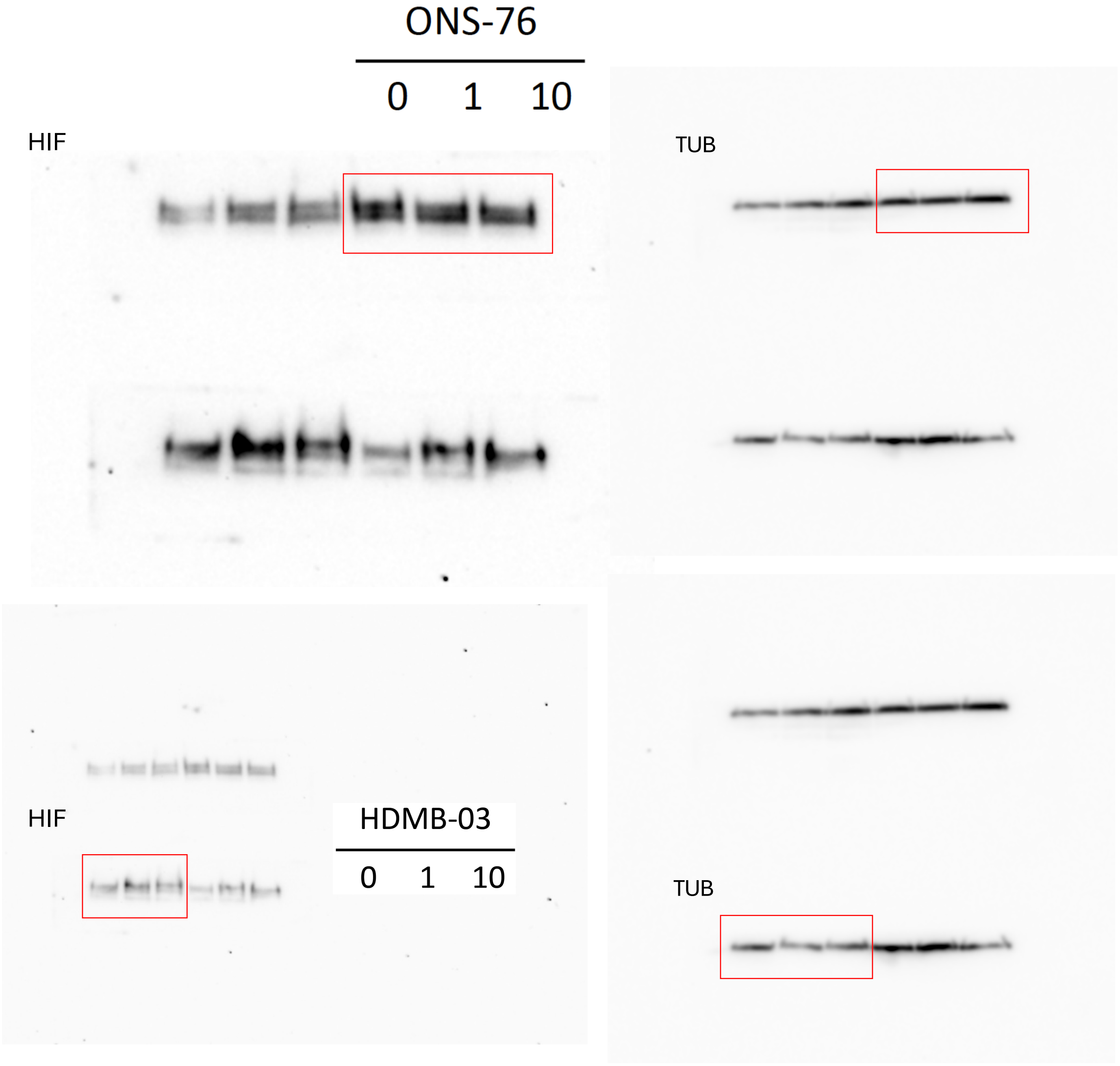

S Figure 6 j

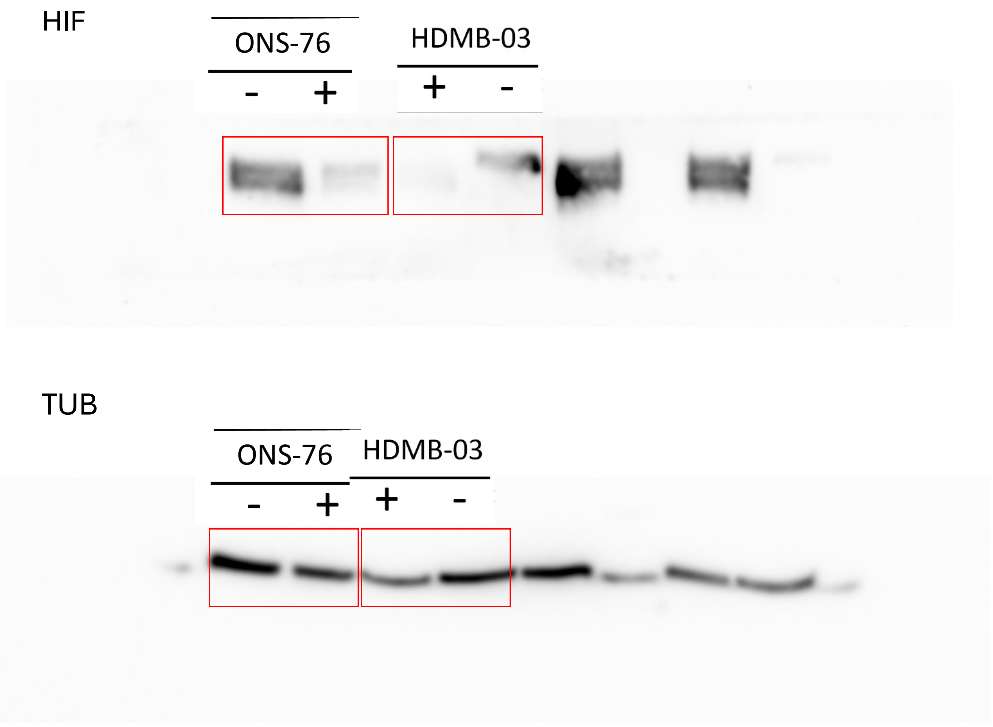

S Figure 6 n

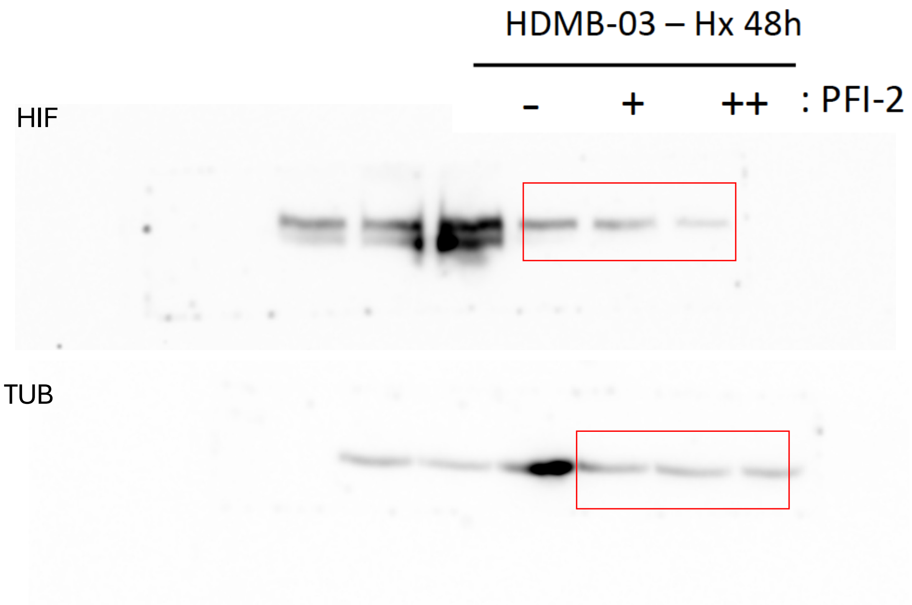

Suppl. Figure 11 a

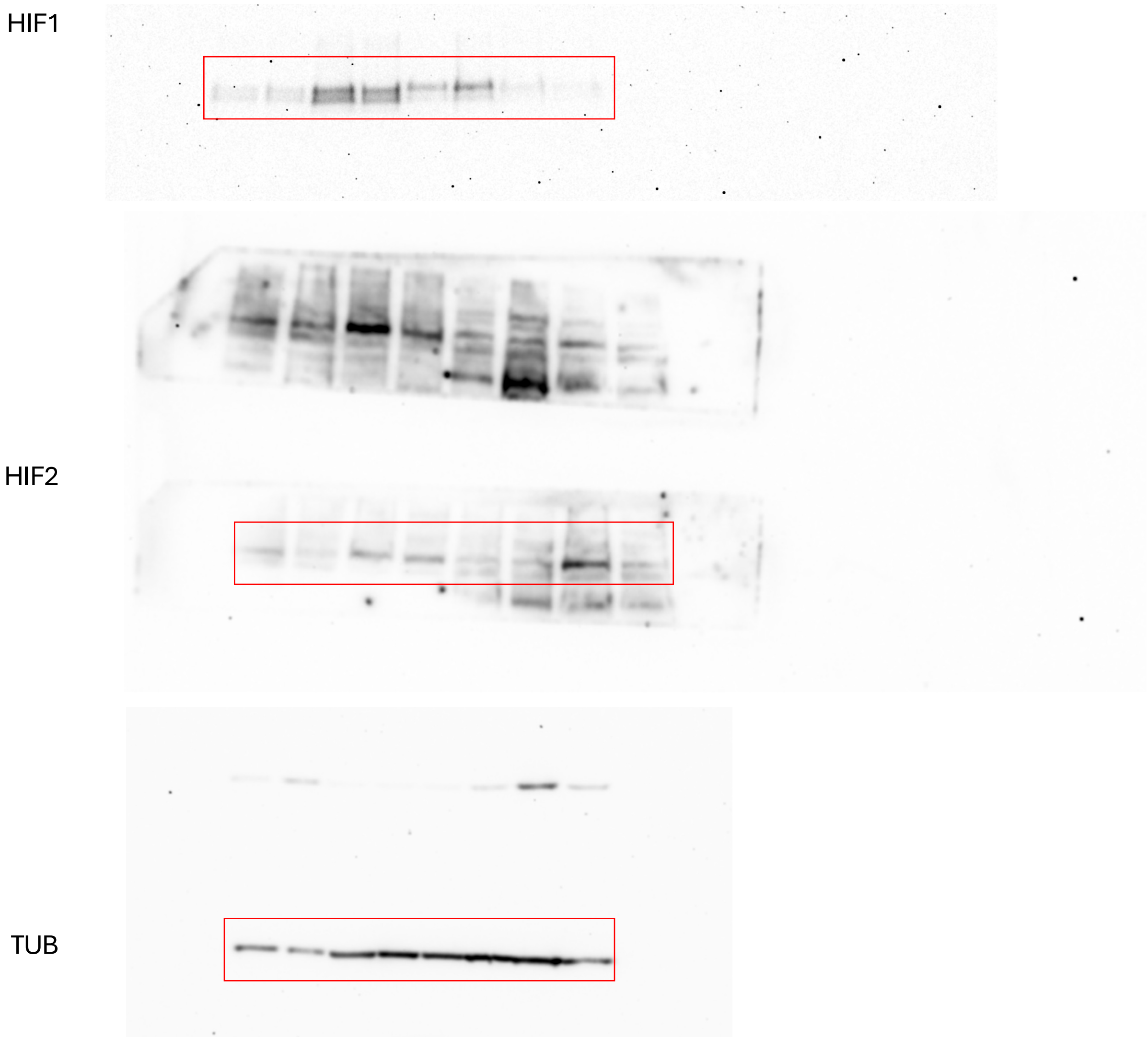

Suppl. Figure 11 b

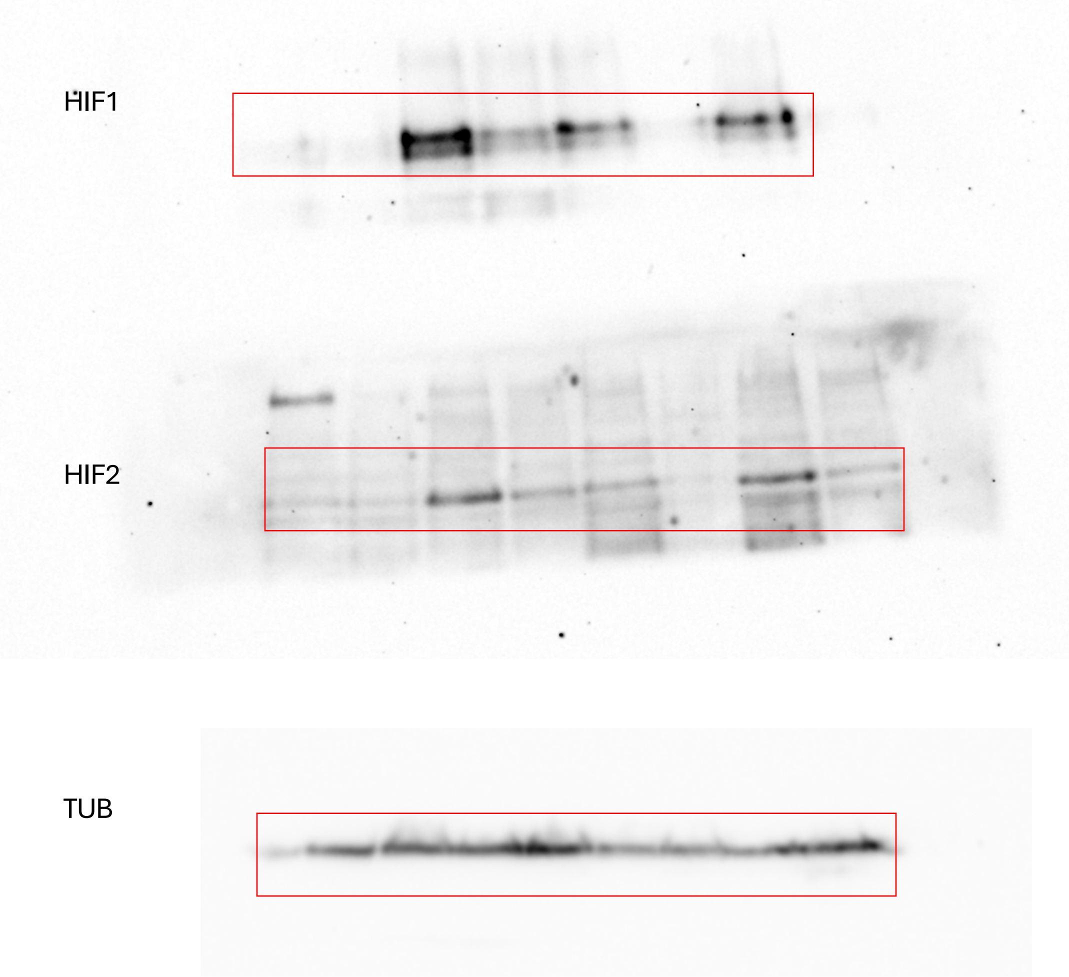

Supplement: Supplementary file 3 — Original data [file 41420_2024_2100_MOESM3_ESM.pdf]
